# Mapping the Evidence: Central Sleep Apnea Syndromes During Sleep and Stroke—A Scoping Review

**DOI:** 10.31083/RN49726

**Published:** 2026-05-26

**Authors:** Karol Uscamaita, Imán Yazbeck Morell, María-José Sánchez-López, Marta García Pla, Olga Parra Ordaz, Adrià Arboix

**Affiliations:** ^1^Neurology Service, Sleep Disorders Unit, Hospital Universitari Sagrat Cor, Grupo Quirónsalud, 08029 Barcelona, Spain; ^2^Medicine Department, University of Barcelona, 08036 Barcelona, Spain; ^3^Department of Internal Medicine, Hospital Universitari Sagrat Cor, Grupo Quirónsalud, 08029 Barcelona, Spain; ^4^Medical Library, Hospital Universitari Sagrat Cor, Grupo Quirónsalud, 08029 Barcelona, Spain; ^5^Family and Community Medicine, CAP La Mina (ICS), 08930 Sant Adrià de Besòs, Barcelona, Spain; ^6^Department of Pneumology, Sleep Disorders Unit, Hospital Universitari Sagrat Cor, Grupo Quirónsalud, 08029 Barcelona, Spain; ^7^Cerebrovascular Division, Department of Neurology, Hospital Universitari Sagrat Cor, Grupo Quirónsalud, Universitat de Barcelona, 08029 Barcelona, Spain

**Keywords:** central sleep apnea, sleep apnea syndromes, stroke, ischemic, sleep-disordered breathing, brain ischemia

## Abstract

**Background:**

Central sleep apnea (CSA) syndromes and acute ischemic stroke are linked by a complex, bidirectional relationship. After ischemic stroke, CSA prevalence rises markedly compared with the general population, in which it is usually below 1%. In stroke cohorts, CSA frequencies of around 8%–12% have been reported, although estimates are heterogeneous owing to differing diagnostic criteria. In this scoping review we map the evidence on CSA syndromes during sleep in patients with stroke, summarizing prevalence, mechanisms, clinical correlates, and prognostic implications.

**Methods:**

The Medline (PubMed), Scopus, Cochrane Reviews, and Web of Science databases were searched for observational and interventional studies published from inception to August 31 2025 in English and Spanish assessing CSA syndromes in adults with ischemic stroke. Two reviewers independently screened records, selected studies, and extracted data on study design, stroke characteristics, CSA definitions, comorbidities, and clinical outcomes; disagreements were resolved by consensus with a third reviewer.

**Results:**

Fifty-five studies including several thousand patients with ischemic stroke were identified. Across studies, post-stroke CSA prevalence ranged from approximately 8% to 12%, clearly exceeding rates in the general population. CSA was often reported in the absence of overt cardiac comorbidities, and patients with CSA tended to have lower body mass index and fewer classic cardiovascular risk factors than those typically described with obstructive sleep apnea. Pathophysiological analyses emphasized disturbed central ventilatory control after cerebral ischemia; however, several studies did not show a consistent association between specific lesion locations and CSA occurrence, suggesting that stroke may unmask CSA in predisposed individuals rather than cause it solely through focal damage. In older adults, CSA appeared as an independent correlate of ischemic stroke and a potential marker of silent cerebral injury or impaired central respiratory regulation.

**Conclusions:**

Available evidence indicates that CSA syndromes during sleep are substantially more frequent in patients with ischemic stroke than in the general population and may be associated with increased cerebrovascular risk and subclinical brain injury. Heterogeneity in CSA definitions, diagnostic protocols, and outcome measures limits firm conclusions. Standardized criteria and adequately powered prospective studies are needed to clarify the mechanistic and prognostic role of CSA in stroke.

## 1. Introduction 

Ischemic stroke remains a leading cause of death and long-term disability worldwide, and its prognosis is strongly influenced by coexisting cardiovascular and respiratory comorbidities [1]. Sleep‑disordered breathing is highly prevalent in patients with acute ischemic stroke and has emerged as a potentially modifiable factor that may affect both early neurological recovery and long‑term vascular outcomes. Within the spectrum of sleep‑disordered breathing, central sleep apnea (CSA) syndromes have received comparatively less attention than obstructive sleep apnea, yet they may be particularly relevant in the cerebrovascular setting because they reflect instability of central ventilatory control rather than upper airway collapse.

Obstructive sleep apnea is characterized by repeated upper-airway collapse during sleep, whereas central sleep apnea reflects reduced or absent respiratory effort due to instability of central ventilatory control. In stroke patients, these two entities may coexist, but they differ in their predominant pathophysiology, diagnostic scoring, and potential management. CSA is characterized by recurrent episodes of absent or markedly reduced ventilatory effort during sleep, leading to cyclical fluctuations in ventilation, oxygen saturation, and autonomic activity [2]. In the general population, CSA is considered uncommon, with a prevalence well below 1%. In contrast, several cohort studies have shown that the frequency of CSA increases substantially after acute ischemic stroke, with reported prevalence estimates in the range of approximately 8–12%, although figures vary depending on diagnostic criteria, recording methods, and timing of the sleep study relative to the index event [3]. This disproportionate rise in CSA burden after stroke, compared with its rarity in community samples, underscores the possibility of a specific pathophysiological link between cerebral ischemia and disordered central control of breathing during sleep.

The mechanisms underlying CSA in the post‑stroke setting appear multifactorial and are not yet fully understood. Experimental and clinical data indicate that lesions involving supratentorial autonomic networks or infratentorial structures, particularly the medulla, can disrupt the integration of chemoreceptor input and the generation of respiratory rhythm, thereby predisposing to ventilatory instability and central apneic events. However, several imaging‑based studies have failed to demonstrate a consistent association between particular lesion locations and the occurrence of CSA, suggesting that ischemic injury may act as a facilitator or trigger in individuals with pre‑existing vulnerability rather than as a single, focal “lesion site” responsible for CSA [3]. This uncertainty regarding lesion topography and causal pathways complicates risk stratification and the design of targeted interventions. Although the included studies support a network-level disturbance of central ventilatory control, they provide little direct molecular or biomarker evidence on neurotransmitter pathways or chemoreceptive signaling in post-stroke CSA.

Clinically, CSA after ischemic stroke has important potential consequences. Observational evidence indicates that the presence of CSA is associated with higher mortality, poorer functional recovery, and an increased risk of cardiovascular complications [4]. These associations are biologically plausible: CSA promotes ventilatory instability, intermittent nocturnal hypoxemia, and surges in sympathetic nervous system activity, all of which may exacerbate ongoing cerebral injury, impair neuroplasticity, and adversely affect cardiac function. Unlike obstructive sleep apnea, CSA in stroke patients often occurs in the relative absence of obesity and with fewer traditional cardiovascular comorbidities such as long‑standing hypertension or diabetes, highlighting that its prognostic impact may not be fully captured by classical vascular risk scores [3]. Importantly, however, current data do not conclusively demonstrate that CSA increases the risk of recurrent ischemic events, and the directionality of the association between CSA burden and stroke outcomes remains incompletely defined.

Management of sleep‑disordered breathing in stroke is an area of active debate. European guidelines encourage systematic screening and treatment of sleep apnea in the acute phase of stroke as part of secondary prevention strategies, whereas American Heart Association and American Stroke Association documents do not mandate routine screening at this stage, reflecting persisting uncertainty about the strength of evidence and feasibility in acute care. Therapeutic decisions depend on the predominant type and severity of sleep apnea. Continuous positive airway pressure (CPAP) is the standard of care for obstructive sleep apnea, but in patients with predominantly central events and high apnea–hypopnea indices, especially in the setting of ventilatory instability without hypercapnia, adaptive servo‑ventilation (ASV) is often considered the preferred modality. In hypercapnic forms of CSA, non‑invasive ventilation may be necessary to provide adequate ventilatory support [3].

Existing interventional studies suggest that treatment with CPAP or ASV in stroke populations is generally safe and may improve daytime symptoms, quality of life, and certain measures of neurological recovery. Nevertheless, the evidence base remains limited, and the impact of treating CSA on long‑term survival, recurrent stroke risk, and broader cardiovascular outcomes is far from settled. Moreover, most clinical trials and observational cohorts have focused primarily on obstructive sleep apnea, leaving CSA and mixed central patterns under‑represented and often analyzed only in small subgroups. As a result, there are currently no specific, evidence‑based recommendations dedicated to the management of CSA syndromes in the context of ischemic stroke, and clinical practice is largely extrapolated from other populations or guided by expert opinion.

In view of these gaps, a comprehensive mapping of the literature on CSA syndromes during sleep in patients with ischemic stroke is urgently needed. A scoping review, conducted according to the PRISMA (Preferred Reporting Items for Systematic reviews and Meta-Analyses) extension for scoping reviews (PRISMA‑ScR), is particularly well suited to capture the breadth and heterogeneity of available evidence, including diverse study designs, varying definitions of CSA and central periodic breathing, and a wide range of clinical and imaging outcomes. The aim of this scoping review is therefore to systematically chart the characteristics, pathophysiological insights, and clinical implications of CSA syndromes after ischemic stroke, and to identify key knowledge gaps that should inform future mechanistic and interventional research.

In the present review, CSA is used as an umbrella term for central apneas and central hypopneas, whereas central periodic breathing and Cheyne–Stokes respiration are treated as descriptive phenotypes that may occur within the broader CSA spectrum

## 2. Materials and Methods

This review was conducted in accordance with the PRISMA‑ScR, following a predetermined protocol agreed upon by all authors (**Supplementary Material-PRISMA checklist**). Institutional review board approval was not required because the study analysed only previously published data. The study protocol was registered with the Open Science Framework (OSF) under the identifier DOI: 10.17605/OSF.IO/H59RJ.

### 2.1 Eligibility Criteria

The objective of this scoping review was to identify and synthesize the available evidence on CSA syndromes in adults with ischemic stroke. The primary research question was: What is the extent of evidence on the prevalence, clinical manifestations, diagnostic methods, management strategies, and outcomes of CSA syndromes in adults with ischemic stroke across different care settings and disease phases?

Population: Studies were eligible if they included adults (≥18 years) diagnosed with ischemic stroke confirmed by clinical and neuroimaging criteria, and if CSA syndromes were reported, identified through polysomnography or equivalent diagnostic testing. Although the prespecified eligibility criterion for CSA relied on a central apnea index threshold, we also retained studies that used pattern-based descriptions of central breathing instability, such as central periodic breathing or Cheyne–Stokes respiration, when they provided relevant CSA-related data. This approach was necessary because the literature uses heterogeneous definitions and reporting standards, and excluding these studies would have omitted clinically informative evidence. Accordingly, the review captures the full spectrum of CSA-related evidence reported after ischemic stroke, while explicitly acknowledging definitional heterogeneity.

Concept: Eligible studies explored the impact of CSA syndromes on stroke patients, including aspects of pathophysiology, diagnosis, treatment, clinical progression, or prognostic significance.

Context: Studies conducted in any healthcare setting (acute hospital units, rehabilitation centers, or community environments) and at any stage of illness (acute <7 days, subacute 1–3 months, or chronic >3 months) were accepted.

All types of quantitative, qualitative, and mixed‑methods research were eligible. These included randomized controlled trials, non‑randomized controlled studies, before‑and‑after or interrupted time‑series designs, prospective and retrospective cohorts, case‑control and cross‑sectional studies, as well as descriptive studies such as case series. Qualitative research using phenomenology, grounded theory, or ethnographic approaches was also accepted. In addition, systematic or scoping reviews meeting the inclusion criteria were considered for inclusion.

Exclusion criteria included:

Studies involving pediatric or non‑human populations.

Editorials, commentaries, and letters to the editor.

Conference abstracts, protocols, or documents without full text.

Papers without specific data on CSA syndromes or without confirmed ischemic stroke populations.

No restriction regarding geographic region was applied.

### 2.2 Information Sources

The literature search covered PubMed (https://pubmed.ncbi.nlm.nih.gov), Scopus (https://www.scopus.com/), Web of Science (Core Collection) (https://www.webofscience.com/wos/woscc/basic-search), and the Cochrane Library (https://www.cochranelibrary.com) databases from inception to 31 August 2025. Searches were restricted to articles published in English or Spanish. An initial pilot search was performed in PubMed to identify key Medical Subject Headings (MeSH) terms and free‑text related to two broad blocks of search terms: “sleep apnea, central” (“periodic breathing”, or “Cheyne-Stokes respiration” or “central breathing disorders”) and ischemic stroke (“brain stem infarctions” or “cerebral infarction” or “brain infarction” or “brain ischemia” or “cerebrovascular disorders”). The final PubMed strategy was adapted for the other databases (**Supplementary Material I**). To ensure completeness, the reference lists of all included papers and relevant systematic reviews were manually examined for additional studies. Grey literature and non‑peer‑reviewed publications were excluded.

### 2.3 Study Selection and Data Extraction

All references retrieved from electronic databases were imported into Zotero 7 for Windows (v. 7.0.22) (Corporation for Digital Scholarship, https://www.zotero.org/) for reference management and duplicate removal. The selection process was conducted in two stages.

First, two independent reviewers screened article titles and abstracts for relevance against the predetermined inclusion criteria. Second, full‑text versions of all potentially eligible studies were obtained and evaluated independently by the same reviewers. Any disagreements were resolved by discussion or consultation with a third reviewer. Data extraction was conducted independently by two reviewers using a customized Microsoft Excel‑based extraction form designed specifically for this review (**Supplementary Material II**). Extracted data included the authorship, year of publication, country, study design, sample size, stroke type, lesion location, phase of disease, diagnostic methods, quantitative respiratory indices (CAI, Cheyne–Stokes respiration percentage, total AHI), comorbidities (atrial fibrillation, congestive heart failure, pulmonary disease), and reported outcomes including neurological severity (NIHSS), functional recovery (mRS, Barthel Index), mortality, and therapeutic interventions (e.g., CPAP or adaptive servo‑ventilation). Any inconsistencies identified during data extraction were discussed and resolved by consensus. Revisions to the extraction form made during the process were documented to ensure transparency.

### 2.4 Data Items

Extracted data were organized according to the following variables:

Study characteristics (design, country, publication year).

Population data and stroke characteristics (phase, lesion location).

Diagnostic procedures applied for identifying CSA syndromes.

Main clinical and neurological outcomes (NIHSS, mRS, mortality).

Reported management and therapeutic strategies for CSA syndromes.

Each article’s main findings were summarized to describe the scope and nature of evidence addressing the predefined research question.

### 2.5 Data Synthesis

Because of the methodological heterogeneity of included studies, a quantitative meta‑analysis was not feasible. Instead, findings were synthesized in a narrative framework and systematically analyzed according to level of evidence, following the hierarchical standards of evidence‑based medicine.

## 3. Results

**Fig. 1.**
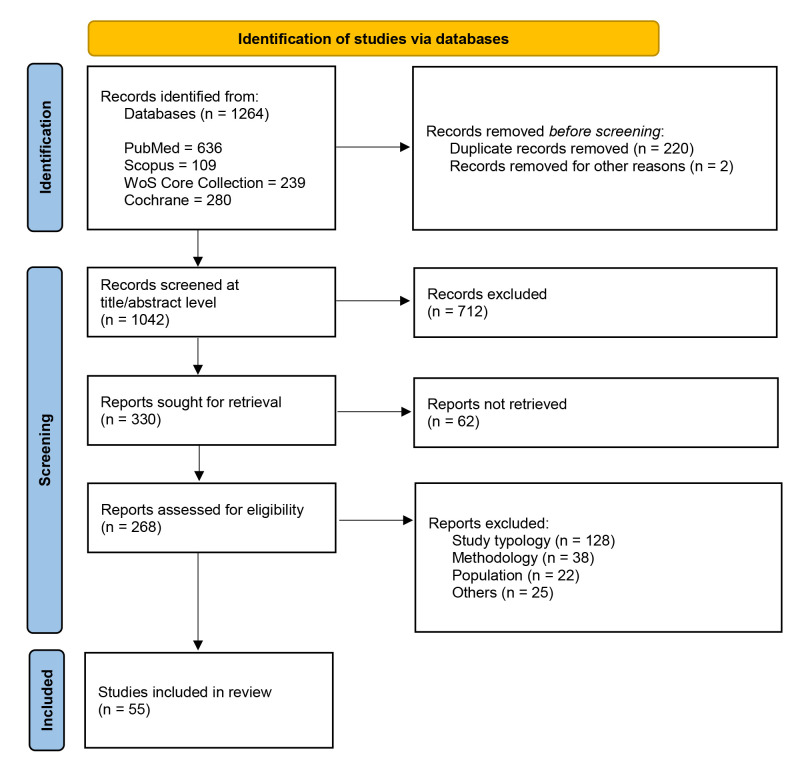
**PRISMA 2020 flow diagram of study selection**. The systematic search across four databases identified 1264 records. Fifty-five studies met final criteria for analysis. PRISMA, Preferred Reporting Items for Systematic reviews and Meta-Analyses.

At the database level, 1264 records were identified (PubMed n = 636, Scopus n = 109, Web of Science Core Collection n = 239, Cochrane n = 280), with 220 duplicate records and 2 additional records removed before screening. After de‑duplication, 1042 records were screened at title/abstract level, and 712 were excluded. Of 330 reports sought for retrieval, 62 could not be retrieved; 268 full texts were assessed for eligibility, and 213 were excluded mainly due to study typology (n = 128), methodology (n = 38), population (n = 22), or other reasons (n = 25). The final scoping review included 55 studies (Fig. 1).

### 3.1 Study Selection and General Characteristics

Across the 55 included studies Table 1 (Ref. [3, 5, 6, 7, 8, 9, 10, 11, 12, 13, 14, 15, 16, 17, 18, 19, 20, 21, 22, 23, 24, 25, 26, 27, 28, 29, 30, 31, 32, 33, 34, 35, 36, 37, 38, 39, 40, 41, 42, 43, 44, 45, 46, 47, 48, 49, 50, 51, 52, 53, 54, 55, 56, 57]), 20 provided original CSA or central periodic breathing (CPB)/Cheyne–Stokes respiration (CSR) data in adult patients with ischemic stroke or mixed stroke cohorts including ischemic stroke [3, 5, 6, 7, 8, 9, 10, 11, 12, 13, 14, 15, 16, 17] to larger observational cohorts (n = 139 [6]; n = 156 [7], 2006; n = 182 [11], 2018; n = 166 [12], 2020) and a population‑based home sleep apnea test (HSAT) cohort of 1346 ischemic stroke patients [17].

**Table 1.** **Characteristics of 55 included studies on central sleep apnea syndromes in ischemic stroke**.

	Author & Year	Study design	Sample size (N)	Stroke phase (acute/subacute/chronic)	Type of sleep study (PSG/polygraphy)	Definition of CSA/CSB (index)	CAI or AHI (central-related)	Main clinical outcomes reported (CSA/CSB-related)	Level of Evidence (OCEBM)
1	Baillieul S *et al*., 2022 [3]	Prospective monocentric cohort; cross-sectional analysis at ≥3 months post–first ischemic stroke	185 first ischemic stroke patients	Chronic (median 134.5 days after stroke onset)	Full-night in-laboratory polysomnography	Central AHI (cAHI); coexisting and central sleep apnea defined by cAHI/AHI ≥20; central sleep apnea by cAHI/AHI ≥50 and AHI ≥5	Central AHI reported (events/h) and proportion of central events (cAHI/AHI)	Prevalence of coexisting/central sleep apnea 42.9% among moderate–severe SDB; higher cerebellar lesion frequency and heightened hypercapnic ventilatory response in coexisting/central SA vs no/mild SDB; association of infratentorial lesions, male sex, obesity and age ≥65 with moderate–severe SDB	Level 1b
2	Parenti A *et al*., 2005 [21]	Case report with neuropathologic examination (two fatal cases)	2 patients	Chronic CSA with acute fatal medullary ischemic–hypoxic lesions; stroke lesions described as acute at death in medullary tegmentum	Diagnostic nocturnal polysomnography before death	Central sleep apnea described by central apneas per hour; no explicit CAI label; CSA defined clinically by central apneas with hypoxia and hyperventilation phases	Case 1: 38 central sleep apneas/hour; Case 2: 35 central sleep apneas/hour	Acute bilateral ischemic–hypoxic lesions of solitary tract nuclei in chronic CSA; suggestion that CSA-related hypoxemia contributes to selective stroke of solitary tract nuclei and that CSA may contribute to stroke pathogenesis via hypoxia/hemodynamic oscillations	Level 4
3	Qu XR *et al*., 2024 [22]	Single-patient case report with detailed diagnostic and PAP titration studies	1 patient	Subacute (sleep study performed about 3 months after acute medullary/bulbar infarction)	In-laboratory nocturnal polysomnography with PAP titration and transcutaneous CO_2_ monitoring	Central apnea–hypopnea index (central AHI); CSA defined as acquired central sleep apnea after medullary infarction with central AHI predominating, occurring almost exclusively in Rapid Eye Movement (REM)sleep	Central apnea–hypopnea index 70.1 events/h (baseline PSG)	REM-predominant CSA with severe desaturations (ODI 50.8/h, nadir SpO_2_ 72%); marked reduction of central events after CPAP 16 cm H_2_O and near resolution with BiPAP ST 17/13 cm H_2_O when PtcCO_2_ maintained above apneic threshold; sustained symptomatic improvement at 6 months of home NIV	Level 4
4	Bassetti C *et al*., 1997 [23]	Prospective observational study of consecutive acute stroke patients	39 patients with first acute ischemic stroke	Acute (polysomnography mean 10 days after stroke onset; range 1–49 days)	Bedside overnight polysomnography	Sleep apnea diagnosed if AHI ≥10; central sleep apnea when ≥50% of events central; Cheyne–Stokes breathing (CSB) defined as periodic breathing with central apneas/hypopneas alternating with hyperpnea in crescendo–decrescendo pattern over ≥10% of total sleep time	AHI in NREM and REM reported; central vs obstructive proportions given; no single CAI but central apnea burden described within CSB and central SA definitions	67% had sleep-disordered breathing; OSA in 54%, CSB in 28% (often coexisting with OSA); abnormal breathing during wakefulness in 18%; prevalence and severity of SDB similar in supra- vs infratentorial strokes; trend to poorer short-term outcome in those with breathing abnormalities, especially CSB and wake abnormalities	Level 2b
5	Power WR *et al*., 1982 [24]	Single-patient case study with two polysomnographic nights	1 patient	Chronic (sleep complaints and CSA dating back 10 years after cerebral infarction)	Full-night polysomnography on two separate nights	Cheyne–Stokes respiration (CSR) characterized as central periodic breathing; no explicit CAI; CSA defined by cyclical central apneas and hyperpneas; analysis by proportion of CSA time in each sleep stage	No numeric CAI; CSA occupied 88% of light NREM and 83% of slow-wave sleep; 2% of REM sleep time showed CSA, 98% regular respiration	CSA predominantly during NREM sleep with near-normal respiration in REM; CSA-linked arousals (≈23/h during CSA vs 8/h with normal breathing) caused severe sleep fragmentation and insomnia; supports concept of separate CNS mechanisms for REM vs NREM respiratory control	Level 4
6	Rowat AM *et al*., 2006 [7]	Prospective observational cohort of acute stroke patients with portable respiratory monitoring and imaging correlation	134 patients with acute stroke	Acute (patients included within 24 h of a suspected first or recurrent ischemic or hemorrhagic stroke)	Validated portable continuous monitoring equipment (Embletta PDS, Medcare Flaga) recording breathing patterns; no full polysomnography described	Central periodic breathing (CPB), including Cheyne–Stokes respiration, defined as cyclical increases in rate and depth of breathing alternating with hypopnea/apnea; no numeric CAI cut-off given	Not reported as CAI or central AHI; CPB presence (yes/no) and proportion of time are described but no explicit CAI/AHI values provided	CPB was associated with large acute cerebral hemispheric lesions, severe mass effect, and previously reported poor functional outcome at 3 months; no association with severity of prior cerebrovascular disease	Level 2
7	Siccoli MM *et al*., 2008 [9]	Prospective observational study of consecutive first-ever acute ischemic stroke patients with nocturnal respirography and cardiac workup	74 patients	Acute (patients admitted within 96 hours after stroke onset; first night after admission studied)	Overnight respirography with a validated portable device (Autoset Embletta PDS, ResMed), not full PSG	CPBS defined as ≥3 cycles of regular crescendo–decrescendo breathing with ≥50% reduction in nasal airflow and respiratory effort lasting ≥10 s; central apnea index (CAI) separately scored	In all patients: central apnea index 7 ± 12/h (0–61); in those with sleep apnea (AHI >10/h): CAI 12 ± 14/h (0–61)	CPBS was frequent (present in 72%; ≥10% recording time in 41%) and its severity was associated with older age, higher stroke severity, ECG abnormalities, and lower left ventricular ejection fraction; more severe in extensive hemispheric strokes and less frequent in left insular and mesencephalic strokes	Level 2
8	Sacchetti ML and Della Marca G, 2014 [25]	Narrative hypothesis/theoretical article proposing a classification of stroke plus SDB phenotypes; not an original patient cohort study	Not applicable (no explicit patient series with N; article is conceptual)	Discusses ischemic stroke in general; acute/subacute/chronic phases are mentioned conceptually but no defined phase for a specific cohort	Refers broadly to polysomnography (PSG) and analysis of sleep microstructure (CAP) in stroke patients, but no single study protocol is reported	Discusses OSA, CSA, Cheyne–Stokes respiration, complex sleep apnea, loop gain and CAP, but does not define CSA/CSB by a specific numeric index such as CAI or central AHI	Not reported; indices such as AHI/CAI are mentioned in cited literature and figures but no explicit central index values are presented for a defined stroke cohort within this paper	Main outcome is a proposed four-subgroup classification of stroke plus SDB phenotypes to help identify potential best responders to CPAP; no primary clinical outcome data (e.g., mortality or functional scores) reported	Level 5
9	De Paolis F *et al*., 2012 [26]	Single-patient case report with serial cardio-respiratory monitoring	1 patient	Acute post-stroke (central sleep apnea documented three days after a cardio-embolic ischemic stroke)	Portable cardiorespiratory monitoring (nasal flow, thoracic/abdominal movements, oxygen saturation, heart rate); not full PSG	Central sleep apnea syndrome with central apnea index; Cheyne–Stokes breathing mentioned in background, but CSA is characterized by central apnea index rather than a separate CSB label	Before stroke: AHI 28/h, central apnea index 3/h; three days after stroke: AHI 70/h, central apnea index 56/h; under BiPAP: AHI 1/h, ODI 2/h	Stroke in a patient with OSAS led to conversion to severe central sleep apnea syndrome, requiring change from CPAP to BiPAP to control central events; illustrates that acute lacunar stroke can unmask or induce CSA in OSAS	Level 4
10	Brill AK, *et al*., 2014 [18]	Single-centre retrospective analysis of ASV treatment for CSA in post-acute ischemic stroke patients without CHF	15 patients with CSA related to ischemic stroke out of 154 ASV-treated patients	Post-acute/chronic: ASV started a median of 11 months after the acute cerebrovascular event; patients with acute stroke <1 month were excluded	Polysomnography or cardiorespiratory polygraphy used to diagnose non-hypercapnic CSA before ASV; follow-up based on ventilator-reported residual events	CSA defined as non-hypercapnic central sleep apnea/ with >50% of apneas/hypopneas central in origin and apnea index (AI) >5/h; AHI used to quantify severity and treatment response	Baseline AHI 46.7 ± 24.3/h vs 8.5 ± 12/h on ASV at 3 months (*p* = 0.001); central fraction >50% of events by inclusion criteria; individual CAI values are not separately tabulated	ASV significantly reduced AHI and tended to reduce Epworth Sleepiness Scale scores, with mean nightly ASV use 5.4 ± 2.4 h at 3 months and benefits maintained at 6 months, indicating good tolerance and clinical effectiveness in persistent CSA after stroke	Level 3
11	Hermann DM *et al*., 2007 [8]	Prospective case series from a cohort (31 acute first-ever stroke patients, 3 with CPBS described in detail)	31 stroke patients total; 3 with CPBS	Acute (polysomnography and respirography within first 10 days, mean 4.6 ± 2.5 days; follow‑up at 1–3 months)	Polysomnography plus respirography (Autoset)	CPBS defined as ≥3 cycles of crescendo–decrescendo breathing with ≥50% reduction in nasal airflow for ≥10 s	AHI used; CPBS patients had predominantly central apneas/hypopneas (e.g., AHI 5, 25, 28/h in acute phase; Autoset central AHI also reported)	Presence of CPBS in unilateral supratentorial/thalamic strokes; association with lesions in insula, cingulate cortex, thalamus; spontaneous partial resolution of CPBS and improvement in AHI and desaturations at 1–3 months	Level 4
12	Rowat AM *et al*., 2006 [7]	Prospective observational cohort study	156 monitored stroke patients; 138 with usable breathing recordings; 33 with CPB	Acute (recording started median 4 h after stroke; within 24 h of onset; follow‑up 3 months)	Portable multi‑channel respiratory monitoring (nasal pressure, thoracic/abdominal bands, pulse oximetry; not full PSG)	CPB defined as regular crescendo–decrescendo pattern with apnea (cessation) or hypopnea (≥50% reduction) 6–10 s, ≥3 consecutive cycles, with central pattern (reduced effort; obstructive events excluded)	No specific CAI; CPB quantified as % of valid recording time with CPB (median 38 min; many ≥10% of recording)	CPB present in 24%; associated with more severe strokes, congestive heart failure, dysphagia, reduced consciousness; independently associated with death or dependency (mRS ≥3) at 3 months (adjusted OR ≈5.9); higher mortality and lower Barthel Index in CPB group	Level 2
13	Rupprecht S *et al*., 2010 [27]	Cross‑sectional prospective study	59 patients with internal carotid artery stenosis: 49 extracranial (eICA), 10 intracranial (iICA)	No clinical stroke phase; asymptomatic carotid stenosis (non‑stroke population)	Standard overnight polysomnography	CSA defined by cAHI ≥5/h with ≥50% of events central (central apnea–hypopnea index, cAHI)	cAHI (central apnea–hypopnea index); relation of cAHI to stenosis severity and HRV parameters reported	CSA occurred in 39% with eICA stenosis, none with isolated iICA stenosis; CSA associated with severe (≥70%) eICA stenosis, asymmetric stenosis (eICA ratio), and autonomic imbalance (↓HF power, ↑VLF, ↑LF/HF); authors propose CSA as marker of autonomic dysfunction and increased cerebrovascular/cardiovascular risk	Level 3
14	Muñoz R *et al*., 2012 [28]	Population‑based prospective cohort study	394 stroke‑free, community‑dwelling subjects ≥70 years at baseline; 20 ischemic strokes during mean 4.5‑year follow‑up	No baseline stroke (primary prevention); incident ischemic stroke during follow‑up in elderly	Fully attended overnight polysomnography	Central apnea index (CAI; number of central events per hour). CSA episodes defined by central apneas (no thoraco‑abdominal movement) with ≥4% desaturation; groups by CAI ≥1/h and ≥3/h	CAI (central apnea index); CAI categories (≥1 or ≥3 events/h) used in Cox models predicting incident ischemic stroke	Higher CAI associated with increased risk of incident ischemic stroke; event‑free survival lowest in highest CAI group; adjusted HR ≈2.65 for CAI ≥1/h (with AF), ≈3.08 for CAI ≥3/h (with AF and sex); obstructive AHI, T90, arousal index not associated with stroke risk	Level 2
15	Schütz SG *et al*., 2021 [17]	Population‑based cohort of ischemic stroke patients with cross‑sectional HSAT assessment soon after stroke	1346 ischemic stroke participants with analyzable home sleep apnea tests	Recent ischemic stroke; HSAT median 13 days after stroke recognition (IQR 6–21 days)	Home sleep apnea test (ApneaLink Plus; limited‑channel polygraphy, not full PSG)	CAI (central apnea index) = central apneas per hour; CSA defined as CAI ≥5/h and CAI/REI ≥0.50 (≥50% of all respiratory events central)	CAI reported (median 0/h overall); CSA prevalence 1.4% (19/1346)	CSA prevalence low (1.4%) after ischemic stroke; CSA more common in males and in non‑obese (normal/overweight) vs obese; no significant association of CSA with NIHSS, heart failure, or most comorbidities; suggests CSA is uncommon after stroke and HSAT may be acceptable when PSG not feasible	Level 2–3
16	Nogueira RC *et al*., 2021 [29]	Single-patient case report (case study)	1	Acute phase “during the acute phase of stroke” and 1 day after admission, NIHSS 20 at onset, thrombolysis 3.5 h after ictus	No polysomnography; respiratory pattern and Cheyne–Stokes respiration assessed with transcranial Doppler, blood pressure Finometer, and end-tidal CO_2_ capnography	Cheyne–Stokes respiration described clinically only no numeric index, no CAI/CSB threshold given	Not reported; only qualitative presence of CSA-related oscillations in cerebral blood flow velocity and blood pressure	CSA associated with large periodic oscillations in cerebral blood flow velocity, blood pressure, and end-tidal CO_2_, with impaired myogenic control (abnormal resistance-area product) in the affected hemisphere, while metabolic control (critical closing pressure) followed CO_2_ changes	Level 4
17	Franklin KA, 2002 [30]	Narrative clinical review (non-systematic) of human studies on cerebral haemodynamics in obstructive sleep apnea and Cheyne–Stokes respiration	Not applicable review article synthesising multiple published studies; no single N	Mixed phases; includes acute, subacute, and chronic post-stroke and heart failure populations from prior studies, but no single phase defined for this review	Various methods summarized from prior work including transcranial Doppler, Xenon‑133 regional cerebral blood flow, SPECT, and polysomnography however, the review does not present a single, unified sleep-study modality or dataset	Discusses central apnea and Cheyne–Stokes respiration conceptually but does not define CSA/CSB by a specific index threshold within this article	Reports example values such as central AHI patterns and cerebral blood flow velocity changes from individual cited studies, but no single CAI/AHI for a defined study cohort is presented in this review	Central apnea with Cheyne–Stokes respiration shows decreased cerebral blood flow velocity during apnea and increased flow after apnea termination, whereas obstructive apnea shows the opposite pattern with potentially hazardous cerebral hypoperfusion after apnea; obstructive sleep apnea is suggested as a potential risk factor for stroke because of repeated nocturnal cerebral hypoperfusion	Level 5
18	Nopmaneejumruslers C *et al*., 2005 [10]	Prospective cohort study of consecutive stroke patients with polysomnography and echocardiography	93 stroke patients	Subacute/early rehabilitation; echocardiography 40 ± 6 days after stroke onset and polysomnography 44 ± 3 days after stroke, in a stroke rehabilitation unit	Full overnight polysomnography with standard scoring; central and obstructive events differentiated using respiratory inductance plethysmography and thoracoabdominal movement patterns	CSA defined as central apneas and hypopneas ≥10 per hour of sleep central apnea–hypopnea index ≥10/h; total sleep apnea defined as AHI ≥10/h	Central AHI 24.7 ± 4.1 events/h in CSA group vs 1.7 ± 0.2 in non‑CSA; total AHI 38.7 ± 5.0 vs 18.1 ± 1.9 events/h (CSA vs non‑CSA)	CSA prevalence 19 in rehabilitation stroke cohort; CSA associated with lower mean nocturnal transcutaneous PCO_2_ (39.5 vs 43.0 mmHg) and a higher prevalence of LV ejection fraction ≤40 (22 vs 5); among CSA patients, those with LV systolic dysfunction (LVEF ≤40) had longer periodic breathing cycle and hyperpnea duration and a Cheyne–Stokes pattern, and hyperpnea duration ≥33 s was highly sensitive and specific for occult LV systolic dysfunction; stroke type and location were not related to CSA	Level 2
19	Hasan F *et al*., 2021 [19]	Systematic review and meta-analysis of observational studies (prospective, retrospective, case–control, cross-sectional) reporting prevalence of sleep disorders after stroke/TIA	64,047 adults from 169 studies 14,032 stroke survivors and 1061 TIA cases in SDB analyses; larger numbers for insomnia and RLS groups	All three phases separate analyses for acute (≤1 month), subacute (1–3 months), and chronic (≥3 months) after stroke/TIA	Sleep-disordered breathing and PLMS diagnosed mainly by in‑laboratory polysomnography (77 studies) or home-based polysomnography (55 studies); insomnia and RLS assessed by diagnostic criteria or questionnaires	Most SDB studies combined obstructive and central sleep apnea; the meta-analysis does not provide a specific CSA/CSB definition or index cut-off (e.g., separate CAI) for central events alone	Pooled prevalence of mild SDB AHI ≥5/h after stroke/TIA acute 66.8, subacute 65.5, chronic 66.2 overall; moderate and severe SDB prevalence also reported, but central-related CAI or AHI values are not separated from obstructive components	Sleep-disordered breathing, insomnia, periodic leg movements in sleep, and restless legs syndrome are highly prevalent throughout acute, subacute, and chronic phases after stroke/TIA; moderate–severe SDB prevalence appears to decrease over time; age, sex, comorbidities, smoking, and region significantly moderate prevalence; findings support importance of systematic screening and treatment of sleep disorders across stroke phases	Level 1
20	Nachtmann A *et al*., 1995 [5]	Prospective observational study of consecutive acute ischemic stroke inpatients with respiratory monitoring and a non-stroke control group	32 conscious patients with acute ischemic stroke and 20 age‑matched controls	Acute phase patients admitted within 3 days after onset of ischemic stroke and monitored while still acute	Bedside respiratory monitoring for ≥1 hour using chest and abdominal strain gauges, finger pulse oximetry, and end‑tidal CO_2_ in 4 patients; no full PSG or polygraphy	Cheyne–Stokes respiration defined as periodic modulation of respiratory motion amplitude >50 of depth occurring >10 times per hour (pattern-based CSA definition, no CAI)	Central-related indices given as frequency of CSA cycles mean modulation frequency 0.7/min; no CAI or central AHI value is reported	CSR present in 17/32 (53%) of acute ischemic stroke patients (59% supratentorial and 40% infratentorial strokes), unrelated to infarct location; CSA associated with periodic drops in arterial oxygen saturation, with mean and minimal SaO_2_ lower than controls, and both intravenous theophylline and low‑flow oxygen inhalation promptly normalized breathing pattern and oxygen saturation without adverse effects	Level 3
21	Hardavella G *et al*., 2006 [31]	Narrative review (physiopathology, clinical data, therapy)	Not applicable (review)	Not specified as a single phase; discusses acute and longer‑term after stroke	Not a primary sleep‑study; summarizes prior work (no single PSG/polygraphy protocol reported)	Discusses Cheyne–Stokes respiration as recurrent central apneas with crescendo–decrescendo tidal volume; no quantitative CAI/AHI cut‑off defined	Not reported as a specific CAI/AHI; only qualitative description of central apneas/Cheyne–Stokes cycles	Cheyne–Stokes respiration described as common after stroke, associated with severe neurological derangement and possibly higher mortality, and considered a marker of poor prognosis especially with coexisting heart failure	Level 5
22	Kim Y *et al*., 2018 [11]	Observational cohort (prospective registry‑based analysis of consecutively admitted acute ischemic stroke patients with sleep test)	182 ischemic stroke patients with analyzable sleep studies	Acute ischemic stroke (onset within 1 week; sleep test at mean ∼9 days after onset)	Portable cardiorespiratory sleep study (limited‑channel polygraphy; no electroencephalogram (EEG)	CSA defined per 2016 AASM v2.3: ≥3 consecutive central apneas/hypopneas in crescendo–decrescendo pattern with cycle length ≥40 s and ≥5 central events/h with CSA pattern recorded ≥2 h (central apnea index implicit)	Central apnea index significantly higher in CSA vs non‑CSA (median CAI 14.2 vs 1.33 events/h)	CSA present in 19.1 of patients; independently associated with higher premorbid mRS, bilateral hemispheric lesions, left‑atrial enlargement (higher LAVI) and left‑ventricular ejection fraction <50; in a subgroup without cardiac disease, only higher premorbid mRS remained independently associated with CSA	Level 2b
23	Mekky J *et al*., 2023 [16]	Case–control study (first‑ever ischemic stroke patients vs age‑matched controls with overnight PSG)	93 ischemic stroke patients and 50 controls	Subacute phase (PSG within 1 week after stroke; NIHSS followed at 1 month)	Full attended overnight PSG	Central sleep apnea reported via “CSA index” and central AHI component; no specific numeric CAI threshold given for CSA definition beyond standard AASM scoring	Central sleep apnea index higher in stroke vs controls (mean CSA index 1.77 vs 0.14 events/h) and AHI higher in cortical than other sites; desaturation index and lowest SpO_2_ worse in sizable infarcts	Stroke patients had worse sleep architecture and higher AHI, CSA index, and desaturation index than controls; cortical strokes showed higher moderate–severe AHI; sizable infarcts had higher limb movement index, higher desaturation index, and lower minimal SpO_2_; lower minimal SpO_2_ at 1 week correlated with worse NIHSS at 1 month	Level 3b
24	Srijithesh PR *et al*., 2024 [32]	Prospective observational longitudinal cohort	111 recruited ischemic stroke patients; 105 with adequate baseline PSG; 56 with follow‑up PSG	Subacute/early chronic: baseline PSG within 6 weeks of ischemic stroke onset; repeat PSG at ∼3–4 months; clinical follow‑up to 24 months	Full overnight level‑1 PSG	Central sleep apnea quantified by central apnea index; SDB defined by total AHI (cut‑offs: AHI ≥5, 5–14, 15–29, ≥30); specific CSA definition beyond CAI ≥5 not separately detailed	At baseline, 7.6 had CAI ≥5; overall 81.9 had AHI ≥5 and 38 had AHI >30; at follow‑up 56.8 still had AHI ≥5 and 12.5 had AHI ≥30, with CAI and AHI generally decreasing over time	High prevalence of SDB after ischemic stroke (mainly obstructive); AHI and arousal index decreased over 3–4 months, with improvements in sleep efficiency and REM sleep; moderate–severe SDB (AHI ≥15) associated with higher risk of recurrent vascular events or death during 24‑month follow‑up (HR ∼4.8 vs no/mild SDB)	Level 2b
25	Johnson KG & Johnson DC, 2010 [33]	Systematic review and meta‑analysis of observational studies	29 studies, 2343 patients with ischemic/hemorrhagic stroke or TIA	Mixed phases (acute to chronic): timing ranged from within 1 week to ∼3 years post‑event across included studies	Mix of full PSG with EEG, limited‑channel studies, and auto‑CPAP diagnostic mode	Central sleep apnea/Cheyne–Stokes reported as “primarily central apnea” proportion and via AHI thresholds; 17 studies reported percentage of patients with primarily central apneas (no uniform CAI cut‑off reported)	Across 17 studies, 7 (95% CI 4.5–12.0) of patients had primarily central apnea; global SDB prevalence: AHI ≥5 in 72 and AHI ≥20 in 38 of stroke/TIA patients	SDB is very common after stroke/TIA (mostly obstructive); only a small proportion have predominantly central apnea; SDB prevalence did not differ significantly by event type, timing after stroke, or monitoring type; higher SDB rates seen in males, recurrent strokes, and strokes of unknown etiology, lower in cardioembolic strokes	Level 2a
26	Huhtakangas JK *et al*., 2022 [34]	Prospective observational cohort of consecutive ischemic stroke patients with sleep study and long-term follow-up	204 ischemic stroke patients with cardiorespiratory polygraphy	Acute (sleep study within first 48 h after symptom onset) with 6-year follow-up	Unattended cardiorespiratory polygraphy (portable type 4 device, ApneaLink Plus)	Central apnea index (CAI, events·h^-1^) used; central sleep apnea defined when >50% of events central; respiratory event index (REI) used for overall SDB	Mean CAI 3.9/h overall; CAI higher in non-survivors (over twofold vs survivors; exact values not separately tabulated); in REI ≥30 subgroup, higher CAI predicted mortality in univariate analysis	Higher CAI, greater nocturnal hypoxemia (longer T90, lower minimum SaO_2_) associated with increased long-term mortality after ischemic stroke, but polygraphy variables including CAI did not predict recurrent ischemic events; CPAP users had fewer recurrent events but numbers were small	Level 2b
27	Hudgel DW *et al*., 1993 [35]	Physiologic comparative study of convalescent unilateral stroke patients vs healthy elderly controls, with detailed respiratory mechanics during sleep	8 stroke patients and 8 age-, sex-, and size-matched healthy elderly controls (respiratory analysis subset)	Subacute/chronic (≥1 month after unilateral stroke, during inpatient rehabilitation)	Full polysomnography with additional upper airway resistance and EMG measurements	Central vs obstructive events differentiated by presence/absence of inspiratory EMG; Cheyne–Stokes–like periodic breathing described; no explicit numeric CAI definition label, but central vs obstructive apneas classified for AHI and pattern analysis	Group mean apnea–hypopnea index 44 ± 12 events/h in stroke patients vs 12 ± 7 events/h in controls; proportion of central vs obstructive events not numerically separated for an index	Stroke patients showed larger oscillations in upper airway resistance and tidal volume during NREM sleep leading to more obstructive apneas/hypopneas and greater oxygen desaturation; periodic/irregular breathing with mechanical (upper airway) component demonstrated in convalescent stroke	Level 3
28	Lisabeth LD *et al*., 2019 [36]	Population-based prospective cohort with repeated sleep studies and GEE modeling	414 first-ever ischemic stroke patients with at least one portable sleep apnea test (total 1656 sleep studies across time points)	Mixed acute/subacute/chronic: baseline at median 5 days after stroke, then 3, 6, and 12 months	Portable home sleep apnea testing (ApneaLink Plus) at hospital or home	Central apnea index (CAI, central apneas·h^-1^) and obstructive apnea index (OAI) as components of respiratory event index (REI, apneas+hypopneas·h^-1^); central apneas defined by absent respiratory effort on pneumatic effort sensor	Baseline mean CAI 1.6 ± 3.1/h; over one year CAI increased in non-Hispanic White patients by 0.14 events/h per month and did not change in Mexican Americans; REI remained stable, OAI increased over time	SDB (elevated REI) was common and remained stable through 12 months; obstructive component worsened slightly, whereas central component increased only in non-Hispanic Whites; ethnic differences in CAI trajectory identified but central events did not remit overall	Level 2b
29	Gregori-Pla C *et al*., 2019 [37]	Pilot physiologic study (prospective case series) of acute ischemic stroke patients with simultaneous diffuse correlation spectroscopy and respiratory polygraphy	28 screened with pulse oximetry; 5 patients with ≥3% SpO_2_ fluctuations and altered breathing patterns included for combined DCS–polygraphy analysis (239 apneas/hypopneas)	Acute (within first 7 days after anterior circulation ischemic stroke; median 4 days)	Respiratory polygraphy (Embletta MPR PG) plus pulse oximetry, simultaneous with near‑infrared diffuse correlation spectroscopy for CBF	Apneas classified as obstructive, central, or mixed by standard polygraphy (absence vs presence of respiratory effort); AHI (events·h^-1^) and ODI3 used; one patient showed predominantly central apneas with Cheyne–Stokes–like periodic breathing; no standalone CAI label reported	Reported AHI values per case: 13–63.8 events/h, including one patient with mainly central apneas and another with obstructive events; central vs obstructive indices not separately quantified beyond pattern description	Individual apneas/hypopneas were associated with biphasic bilateral microvascular CBF changes (≈27–29% increase then ≈19–21% decrease) and concurrent HR increase and SpO_2_ fall; patients with highest AHI (including central pattern) had most severe strokes and poor 3‑month outcome (two deaths), suggesting potential detrimental hemodynamic impact of CSA/CSB in acute stroke	Level 4
30	Kario K *et al*., 2004 [38]	Single-patient case report	N = 1 (67‑year‑old man with congestive heart failure, carotid occlusion, and nocturnal ischemic stroke)	Chronic cerebrovascular disease with new nocturnal-onset ischemic stroke; sleep study performed about 1 month after the episode	Nocturnal pulse oximetry plus full polysomnography	Central apnea–dominant sleep-disordered breathing documented: polysomnography AHI 55/h with 71% central apneas (explicit central predominance of Cheyne–Stokes–type breathing)	AHI 55 events/h, 71% central; nocturnal pulse oximetry showed 29.2 episodes/h of ≥4% desaturation vs 4.8/h three months earlier; mean awake SaO_2_ 97%	Newly developed central sleep‑disordered breathing with frequent desaturations temporally associated with nocturnal-onset ischemic stroke in a patient with congestive heart failure and carotid occlusion; authors propose nocturnal hypoxemia and blood pressure reduction as triggers of nocturnal ischemic events in patients with marginal cerebral perfusion	Level 4
31	Javaheri S *et al*., 2017 [39]	Narrative review of observational and interventional studies	Not applicable (review)	Mixed; includes chronic heart failure and post‑stroke populations, not confined to a single stroke phase	Full polysomnography and limited-channel studies are described, but no single uniform protocol; multiple modalities across cited studies	CSA and Hunter–Cheyne–Stokes breathing generally defined by central apneas and periodic breathing with apneic threshold physiology rather than a single numeric CAI/AHI cut-off	Central AHI values vary across cited studies a typical example is baseline AHI ≈40 events/h with CSA/HCSB predominance in CANPAP and SERVE‑HF, but no single central index is defined for all cohorts	In stroke and other cerebrovascular disease, CSA/HCSB is associated with hyperadrenergic state and may improve with PAP or ASV central-event suppression improved sympathetic markers and ejection fraction in heart failure cohorts, while post‑stroke CSA tends to decrease over time unless driven by comorbid heart disease	Level 3–4
32	Stevens D *et al*., 2018 [40]	Narrative mini‑review	Not applicable (review)	Covers acute and chronic post‑stroke phases; notes SDB is more severe in acute phase and persists in >50% at ≥1 month	Both full polysomnography and respiratory polygraphy are discussed wide use of polygraphy in stroke cohorts is emphasized	CSA generally defined by central apneas ≥5 events/h and by predominance of central events within AHI authors distinguish CSA vs OSA but do not fix a single CAI threshold beyond “5 respiratory events/hour”	Reports that stroke cohorts have higher prevalence of CSA ≥5 events/h than non‑stroke SDB cohorts, but no single pooled CAI/AHI‑central value is given	CSA after stroke is common but its specific impact on functional recovery, mortality and lesion‑location relationships remains unclear limited data suggest central events often decrease over months, and ASV for post‑stroke CSA has only retrospective support without outcome data	Level 3–4
33	Riglietti A *et al*., 2021 [15]	Prospective physiological cohort study with 3‑month follow‑up	70 screened; 60 analyzed with baseline and 3‑month follow‑up sleep studies	Acute (within 10 days of onset; median 3.4 ± 1.4 days) and subacute/chronic at 3 months post‑stroke	Respiratory polygraphy (Nox T3 cardiorespiratory monitoring; no EEG)	CSA defined by “dominant central sleep apnea (d‑CSA)” when ≥70% of events are central and central AHI AHIc ≥5/h periodic breathing/Cheyne–Stokes breathing when CSA occupies ≥10% of total time	In acute phase, d‑CSA group had mean central AHI AHIc 27.5 ± 15.1/h and AHI 31.9 ± 15.7/h overall central AHI decreased significantly at 3 months (from 9.4 ± 12 to 4 ± 5.3/h across the cohort)	Central patterns including CSA and mixed CSA/OSA improved substantially over 3 months, whereas obstructive patterns remained frequent CSA was associated with distinct autonomic profile (higher baroreflex gain and ANSI in d‑CSA without hypertension and in infratentorial lesions), suggesting different autonomic adaptations vs OSA	Level 2b
34	Martínez‑García MA *et al*., 2004 [6]	Prospective observational cohort	139 patients with acute ischemic stroke	Acute: polysomnography within 72 hours of symptom onset (mean 1.4 ± 0.8 days)	Limited‑channel respiratory polysomnography with Autoset Portable Plus II; automated AHI plus manual scoring of apnea type	Central apneas defined by absence of thoracoabdominal movement on belt; Cheyne–Stokes breathing defined as characteristic crescendo‑decrescendo central pattern for >10% of recording time	Central apnea index (AIc) 2.4 ± 2.3/h overall central AI 2.9 ± 2.2/h in documented cerebrovascular accidents vs 1.9 ± 2.2/h in TIAs central AI 3.2 ± 1.8/h in nocturnal strokes vs 1.7 ± 1.5/h in daytime strokes	Central events and Cheyne–Stokes breathing were uncommon (5 patients with CSA) compared with obstructive events; nocturnal strokes had higher AHI and more obstructive apneas, supporting pre‑existing OSA as a stroke risk particularly for night‑onset events	Level 2b
35	Liu X *et al*., 2021 [20]	Systematic review and meta‑analysis of observational cohorts	75 studies; 8670 stroke patients in pooled analysis	Mixed phases; stroke phase categorized as acute (≤1 month), subacute (1–3 months), chronic (>3 months) for subgroup analyses	Type 1 attended full PSG, type 2 unattended full PSG, and type 3 devices; only studies using PSG (types 1–3) included	CSA defined as central events making up ≥50% of respiratory events in AHI; central sleep apnea prevalence calculated as proportion with predominantly central events CSA ≥50% central	Central sleep apnea prevalence 10.0% (95% CI 6.5–14.9) among stroke patients obstructive events accounted for 53.1% of cases overall; central prevalence estimates pooled across different CAI cut‑offs and definitions	Central sleep apnea is less common than obstructive events but present in a substantial minority; prevalence varies with stroke subtype (higher in hemorrhagic, supratentorial, cardioembolic) and phase, but heterogeneity remains high and central vs obstructive patterns are not consistently separated in outcomes	Level 2a
36	Folgueira A *et al*., 2021 [14]	Descriptive, cross‑sectional, retrospective study of hospitalized acute ischemic stroke patients with nocturnal PSG	53	Acute (PSG within first 7–10 days after stroke onset)	Full‑night polysomnography with oximetry (PSG nivel 1 and 2)	Central apneas defined and quantified; report “índice de apneas centrales” and tendency to more central apneas in infratentorial lesions (index used CAI/central apnea index)	Central apnea index reported (Índice de apneas centrales); global AHI mean 24.5 ± 20.5, AHI in REM 28.39 ± 26.9; higher number of central apneas and lower minimal SpO_2_ in infratentorial strokes	High prevalence of SDB/OSA (84.9%) in acute ischemic stroke; infratentorial lesions showed lower minimal oxygen saturation and greater tendency to central apneas, suggesting more severe respiratory compromise in these locations	Level 3
37	Ott SR *et al*., 2020 [12]	Prospective, multinational, multicentre observational cohort with full‑night PSG at baseline and 3‑month follow‑up	166 with baseline PSG; 105 with both baseline and 3‑month PSG	Acute stroke/TIA with PSG within first 7 days; follow‑up at 3 months (chronic phase)	Attended full‑night polysomnography (PSG) scored by AASM 2012 criteria	SDB defined as AHI ≥5/h; SDB considered central if ≥50% of events central; central apnea index (CAI) reported	Mean AHI 21.4 ± 17.6/h at baseline decreasing to 18 ± 16.4/h at 3 months; CAI reported (e.g., 2.8 ± 7.7 vs 1.9 ± 5.8/h when TIA excluded); CSA prevalence 13–17%; CSR present in 21–25% and decreasing over time	High prevalence and persistence of SDB after acute stroke/TIA; CSA more often severe and associated with higher AHI and ODI; AHI at baseline predicted worse functional outcome (mRS ≥3) at 3 months; CSA tended to improve more than OSA over time, suggesting partial stroke‑related origin of central events	Level 2
38	Pavšič K *et al*., 2020 [13]	Prospective observational cohort with serial PSG in acute ULMI and follow‑up	28	Acute and subacute (PSG at ∼1–4, 5–10, 14–21 days; follow‑up at 3–6 months)	Polysomnography (portable PSG in ward during acute phase, full PSG in sleep lab at follow‑up)	Central sleep apnea defined by central AHI (cAHI) ≥5/h and obstructive AHI <5/h; mixed CSA/OSA defined when both central and obstructive AHI ≥5/h; central events also quantified as CAHI‑WK during wakefulness	Median acute‑phase AHI 16.5 (5.5–43.3); cAHI 11.0 (3.3–29.1); CAI 8.3 (2.2–23.8) events/h; at follow‑up AHI and cAHI significantly decreased (*p* = 0.007 and *p* = 0.003) while obstructive AHI did not change	CSA was very common in acute ULMI (CSA alone in 43%, plus additional patients with mixed patterns); central events frequently occurred in NREM sleep and during wakefulness; central indices improved over months, suggesting stroke‑induced instability of respiratory control with partial recovery	Level 2
39	Noradina AT *et al*., 2006 [41]	Cross‑sectional, prospective study of recent ischemic stroke patients with portable sleep study (type 4)	28	Recent/acute–subacute (sleep studies within 1–4 weeks after stroke onset)	Limited portable monitoring (ResMed Autoset Portable II; airflow and SaO_2_ only, no EEG/EMG; corresponds to respiratory polygraphy)	SDB defined by AHI ≥5/h; CSA/CSB not specifically separated because thoraco‑abdominal effort was not measured; no explicit CAI definition in the text	AHI used as global index; mean AHI 17.5 ± 11.4; prevalence of SDB: 92.8% (AHI ≥5), 78.5% (≥10), 44.8% (≥15), 37.7% (≥20)	Very high prevalence of SDB after recent ischemic stroke; diabetes mellitus and smoking were independent predictors of significant SDB (AHI ≥15), whereas hypertension and other vascular risks were not; typical SDB symptoms (sleepiness, snoring history) were poor predictors	Level 3
40	Lee MC *et al*., 1976 [42]	Prospective observational study of respiratory patterns in acute brainstem infarction using impedance pneumography and arterial blood gases	23	Acute (patients admitted within 72 hours of symptom onset, monitored during first 2 weeks)	Impedance pneumography with chart recording of respiratory pattern; not a PSG or standard polygraphy (no sleep staging, no AHI/CAI)	Central pattern disturbances defined clinically as Cheyne–Stokes respiration (CSR) and Cheyne–Stokes variant (CSV), cluster breathing, tachypnea; no numerical CAI/AHI indices	No CAI/AHI reported; patterns (CSR/CSV/tachypnea) described qualitatively and related to lesion size and bilaterality; blood gases showed respiratory alkalosis with CSR and tachypnea	CSR and CSV occurred frequently in acute brainstem infarction; prominent CSR or sustained tachypnea were associated with extensive bilateral pontine lesions and poor prognosis (high mortality), whereas normal/CSV‑predominant patterns with unilateral lesions had better outcomes	Level 3
41	Hermann DM and Bassetti CL, 2003 [43]	Narrative review/opinion statement (Current Treatment Options in Neurology)	Not applicable	All phases discussed; includes acute and 3‑month follow-up data from cited studies	Describes respiratory polygraphy for acute stroke; polysomnography for complex cases	Refers to “central sleep apnea” and “Cheyne–Stokes breathing (CSB)” but no single numeric CAI threshold; SDB severity mainly by AHI	Reports SDB prevalence using AHI ≥10/h and ≥25/h; no specific pooled central AHI reported	Suggests CSB and central events improve with oxygen or theophylline; CPAP or other ventilatory support may suppress central apnea/CSB and improve oxygenation in acute ischemic stroke cases	Level 5
42	Baillieul S *et al*., 2022 [44]	Narrative review in Lancet Neurology	Not applicable	Explicitly covers acute (<1 month), subacute (1–3 months), and chronic (>3 months) post‑stroke phases via cited meta‑analyses	Discusses polysomnography (type 1 and 2) as gold standard and respiratory polygraphy (type 3 and 4) as alternative screening/diagnostic tools	Defines central sleep apnea as ≥5 central events/h with central AHI ≥50% of total AHI; Cheyne–Stokes breathing described as periodic central pattern but not defined by a separate index	Summarises central sleep apnea prevalence 8–12% using central AHI criteria (central AHI ≥50% of total AHI, AHI >5/h) across included studies	States that evidence on CSA effects on mortality and functional outcome after stroke is scarce and inconsistent, but suggests central events may persist and require re‑evaluation; focuses clinical outcome data mainly on obstructive events	Level 2
43	Tanayapong P and Kuna ST, 2021 [45]	Clinical review (narrative review) in Sleep Medicine Reviews	Not applicable	Reviews SDB before stroke (risk) and after stroke; includes acute and chronic phases but not restricted to a single phase	Focuses on in‑laboratory polysomnography as diagnostic standard; mentions home sleep apnea testing as possible tool	Defines SDB with AHI; distinguishes OSA vs CSA by >50% obstructive vs central events; CSA frequency given (CSA <10% after stroke) but no single unified CSA index cut‑off beyond AHI‑based definition	Provides pooled data that CSA occurs in <10% of adults after stroke and describes AHI thresholds (e.g., AHI >5, ≥15, ≥30); no single central AHI summary separate from this	Concludes CSA data are too sparse to firmly link CSA to incident stroke or to clear recurrence/mortality risk, whereas SDB/OSA clearly worsen neurological recovery and increase recurrence and mortality; CSA impact remains uncertain	Level 2
44	Alexiev F *et al*., 2018 [46]	Narrative review (Journal of Thoracic Disease)	Not applicable	Addresses SDB both as risk factor and as consequence of stroke; considers acute and longer‑term evolution of SDB after stroke	Refers broadly to sleep studies (apnea–hypopnea index from polysomnography or respirography); specific modality varies across cited work, not a single uniform method	Notes that CSA/CSB are frequent in early days after stroke and linked to older age, stroke severity/extension, and LV dysfunction; does not set a numeric CSA/CSB index definition beyond general AHI use	Summarises that one‑third of stroke survivors have AHI >30/h and that CSA frequency varies by recording interval; central‑specific CAI values are not pooled	States that both OSA and CSA appear to increase long‑term mortality risk in stroke patients and that early CSB/CSA correlate with worse severity and LV dysfunction, but quantitative CSA‑specific outcome effects remain limited	Level 5
45	Plomaritis P *et al*., 2023 [47]	Single-center prospective observational longitudinal study	130 acute stroke patients (110 ischemic stroke, 20 intracerebral hemorrhage)	Acute stroke (polysomnography within 72 h from symptom onset; 3‑month functional follow‑up)	Type 2 unattended overnight polysomnography with full EEG/EOG/EMG, airflow, respiratory effort, oximetry, position, ECG	SDB defined as AHI ≥5; CSA vs OSA defined if >50% of events are central vs obstructive; central apnea identified by absent respiratory effort; CSB not given a separate numeric index	Mean AHI 33.5 ± 24.8; among SDB, 19.4% had central apnea; no separate CAI mean reported, only central vs obstructive classification	CSA presence (vs OSA) strongly associated with congestive heart failure (OR 18.295 for central apnea detection); higher AHI and higher NIHSS independently predicted worse 3‑month functional outcome (mRS 0–1 vs 2–6), indicating SDB severity adversely affects recovery, while central vs obstructive type itself was not an independent outcome predictor	Level 2
46	Bonnin-Vilaplana M *et al*., 2009 [48]	Prospective observational cohort of consecutive lacunar stroke patients	68 (with radiologically proven lacunes)	Acute (respiratory study within first 48–72 h after stroke onset)	Respiratory polygraphy (portable respiratory recording device, validated vs PSG)	Central apnea index (CAI); Cheyne–Stokes respiration defined as periodic breathing with central apnea/hypopnea ≥10% of time in bed	20.6% of patients had CAI ≥5; mean CAI 5.6–8.9 events/h in higher-AHI groups (exact subgroup means reported)	Cheyne–Stokes respiration present in 20.6% of acute lacunar stroke patients; higher CAI and Cheyne–Stokes respiration associated with higher AHI; smoking and capsular/pontine lacunes associated with higher SRBD burden; no clear short-term outcome difference, but recommendation that smokers with capsular/pontine lacunar stroke be screened for SRBD	Level 2
47	Pajediene E *et al*., 2020 [49]	Prospective observational study with questionnaire screening and PSG in a selected subgroup	66 total; 13 underwent PSG	Acute (patients examined 3–10 days after first stroke symptoms)	Full-night PSG with video (portable system, AASM scoring)	Central sleep apnea defined as ≥5 central apneas and/or central hypopneas per hour and central events ≥50% of all apneas/hypopneas; RDI, RERA, PLM indices also used	Central apnea index not given as a mean; CSA present alone in 1 patient and combined OSA+CSA in 2; AHI used for OSA severity, but central-related summary limited to presence/absence	In this highly symptomatic acute stroke subgroup, 12/13 had a sleep disorder: 1 CSA, 2 OSA+CSA, several with PLMD and/or RBD and insomnia; non-breathing disorders (PLMD, RBD, insomnia) were as frequent as breathing disorders; no significant association between stroke type/location and PSG measures; findings support routine screening beyond sleep apnea alone	Level 3
48	Patrizz A *et al*., 2023 [50]	Experimental animal study (mouse model; randomized to MCAO or sham, with physiological and behavioral testing)	Not expressed as human N; multiple groups of young and aged mice (numbers per group given for each experiment, e.g. stroke n ≈ 14–17, sham n ≈ 10–11 in key analyses)	Experimental post-stroke period (post-ischemic days 3–42; translation to acute/chronic human phases not directly defined)	Whole-body plethysmography (not PSG) for breathing; no classical human sleep study, but apnea/Cheyne–Stokes–like patterns quantified	Apnea frequency (apneas/min) used as main index; Cheyne–Stokes–like periodic breathing pattern described; CO_2_ and hypoxic ventilatory responses measured but no CAI label	Apneas markedly increased after MCAO; minute ventilation reduced (hypoventilation) with Cheyne–Stokes–like pattern; severity of apneas correlated with progressive cognitive decline (Barnes maze, fear conditioning); distal MCAO produced apnea and cognitive deficits only in aged mice; chemoreceptor gain largely preserved, suggesting network/plant-gain mechanisms	Not reported	Level 5
49	Mayer-Suess L *et al*., 2024 [51]	Narrative review of human and animal studies (non-systematic literature review)	Not applicable (review article)	Covers acute and chronic phases (risk before stroke and post-stroke evolution; not limited to one phase)	Summarizes multiple modalities (PSG, polygraphy, questionnaires, experimental recordings) from prior studies; no primary sleep study performed	Discusses CSA and Cheyne–Stokes breathing conceptually (e.g., CSA after brainstem stroke, Cheyne–Stokes breathing as primary CSA subtype), but no single operational index defined in this paper	Reports that CSA is an independent stroke risk factor, often improves with recovery but is associated with poorer prognosis; central events frequently seen after brainstem stroke and in Cheyne–Stokes breathing, but detailed pooled CAI/AHI data not primary focus	Not reported	Level 5
50	Cai H *et al*., 2021 [52]	Narrative review on management (non-systematic; includes tabulated RCTs and cohort data)	Not applicable (review article)	Discusses all phases (acute and chronic post-stroke as well as pre-stroke risk)	Summarizes prior PSG-based and polygraphy-based trials (especially CPAP and other interventions); no new sleep recordings	CSA and Cheyne–Stokes breathing discussed as central apnea syndromes; mentions apnea–hypopnea index and central apnea index conceptually, but no new operational definition introduced	Describes CSA as having lower prevalence than OSA but common in cardiac/cerebrovascular disease; notes that CSA is an independent risk factor and negatively correlated with prognosis; mentions that CPAP/BiPAP may not optimally control CSA and that ASV or mechanical ventilation may be more effective in some post-stroke CSA phenotypes	Not reported	Level 5
51	Parra O *et al*., 2000 [53]	Prospective cohort with repeated measures (acute and 3‑month follow-up)	161 (acute phase); 86 re-studied at 3 months	Acute (48–72 h after admission) and stable at 3 months (chronic/subacute)	Unattended portable respiratory recording (respiratory polygraphy) validated against PSG	Central apnea index (CAI); Cheyne–Stokes breathing (CSB) defined as periodic breathing with central apnea/hypopnea in crescendodecrescendo pattern >10% of bedtime	Acute: CAI 5.6 ± 10.1 overall; lower CAI in TIA (3.3 ± 7.9) than hemorrhagic stroke (11.1 ± 15.1). Stable: CAI decreased to 3.3 ± 7.6 overall	High prevalence of SRBD (71.4% with AHI ≥10); central events and CSB frequent acutely (CSB 26.1%) and decreased at 3 months (CSB 7.3%), suggesting central apneas/CSB are largely a consequence of acute cerebrovascular disease rather than pre-existing	Level 2
52	Huhtakangas JK *et al*., 2020 [54]	Prospective observational single-center study	204 ischemic stroke patients (final analyses)	Acute ischemic stroke, sleep study within 48 h after onset of stroke symptoms	Nocturnal unattended portable 3‑channel cardiorespiratory polygraphy (type 4 device, ApneaLinkPlus)	Central apnea index (CAI); central sleep apnea (CSA) defined as ≥50% of events central; mixed apnea index (MAI) also used	Automatically analyzed CAI 0.57 ± 2.2 h^-1^ vs manually scored CAI 3.9 ± 8.0 h^-1^; ICC for CAI 0.440 (poor reliability)	Manual scoring identified CSA (≥50% central events) in 19.1% and obstructive sleep apnea in 80.9%; automatic scoring underestimated severity and missed 18.6% of SA cases and over half of moderate–severe SA; poor agreement for central and mixed apneas (low ICC), implying limited reliability of automatic scoring for CSA characterization in acute stroke	Level 2
53	Schütz SG *et al*., 2022 [55]	Population-based observational cohort with repeated cross-sectional sleep studies over time	1215 stroke participants with home sleep apnea testing; 2811 with Berlin Questionnaire	Post-ischemic stroke (tested within 30–45 days; early subacute phase)	Home sleep apnea test with ApneaLink Plus (nasal pressure, oximetry, respiratory effort; manually reviewed polygraphy)	Central apnea index reported; central sleep apnea defined via central apnea index within REI, but no explicit syndrome threshold (used REI ≥10/h for SDB)	Median central apnea index 0 (IQR 0–2) events/h; SDB defined as REI ≥10/h; mean REI increased from 18.7 ± 15.7 to 22.9 ± 18.1 over 10 years	Prevalence and severity of SDB after ischemic stroke increased over 2010–2019 (SDB from 61% to 76%; REI +0.56 events/h per year); central sleep apnea remained uncommon, indicating trends driven mainly by obstructive events rather than CSA/CSB	Level 2
54	Brunner H, 2008 [56]	Preliminary open-label trial (case series with pre–post comparison; non-randomized, uncontrolled)	10 stroke inpatients with sleep apnea (9 male, 1 female)	Subacute/chronic stroke: mean duration of illness before first PSG 52.6 ± 11.4 days after stroke	Full overnight polysomnography (2200–0600)	Respiratory disturbance index (RDI) includes obstructive, central and mixed apneas plus hypopneas; central apnea index and mixed apnea index (RDI central, RDI mixed) reported, but no explicit CSA/CSB syndrome definition beyond indices	Baseline central apneas 12.4 ± 4.6 events/h; mixed apneas 3.4 ± 1.2 events/h; with mirtazapine, central apneas increased over time overall (e.g., 14.9 ± 5.0 then 16.6 ± 8.1; in non-responders central and mixed apneas rose markedly)	Mirtazapine improved sleep efficiency and reduced RDI by ∼50% in responders but increased total RDI by ∼50% in non‑responders; in non‑responders, central and especially mixed apneas increased, suggesting mirtazapine may worsen central/mixed sleep apnea in some stroke survivors, warranting monitoring of breathing indices (e.g., ODI/RDI) during treatment	Level 4
55	Huhtakangas JK *et al*., 2018 [57]	Prospective cohort with 6‑month follow-up (thrombolysis vs no thrombolysis groups)	204 ischemic stroke patients at baseline (110 with thrombolysis, 94 without); 177 with follow-up sleep study (98 thrombolysis, 79 non-thrombolysis)	Acute ischemic stroke at baseline (sleep study during initial hospitalization); 6‑month post‑stroke follow-up (chronic phase)	Unattended 3‑channel type 4 cardiorespiratory polygraphy (ApneaLink Plus) both at baseline and 6 months	Central apnea index (CAI); obstructive apnea index; mixed apnea index; sleep apnea defined by REI ≥5/h; no separate CSA syndrome threshold beyond central apnea index	Baseline central apnea index 3.6 ± 7.1 events/h overall (4.0 ± 7.6 thrombolysis; 3.1 ± 6.4 non-thrombolysis); at 6 months CAI increased by 2.2 events/h to 5.8 ± 9.5 overall, with increases in both groups (thrombolysis +2.0; non-thrombolysis +2.5, both *p * < 0.05)	Sleep apnea prevalence remained very high at 6 months (92.7%); obstructive apneas declined while central apneas increased in both groups; in the non-thrombolysis group, risk of new sleep apnea diagnosis at 6 months was 6.1‑fold higher vs thrombolysis; thrombolysis and CPAP together predicted decline in REI, and thrombolysis was independently associated with lower incidence of new post‑stroke sleep apnea; central events increased despite stable high overall prevalence, highlighting evolving pattern from obstructive to more central events over time	Level 2

CSA, central sleep apnea; CPB, central periodic breathing; CSR, Cheyne–Stokes respiration; SDB, sleepdisordered breathing; OSA, obstructive sleep apnea; AHI, apnea–hypopnea index; CAI, central apnea index; cAHI, central apnea–hypopnea index; PSG, polysomnography; HSAT, home sleep apnea test; TIA, transient ischemic attack; LVEF, left ventricular ejection fraction; NIHSS, National Institutes of Health Stroke Scale; mRS, modified Rankin Scale; BMI, body mass index; TOAST, Trial of Org 10172 in Acute Stroke Treatment; CPBS, central periodic breathing during sleep; PLMS, Periodic limb movements during sleep; RLS, restless leg syndrome; OCEBM, the Oxford Centre for Evidence-Based Medicine, Levels of Evidence framework; ↓, decrease in value; ↑, increase in value.

Most CSA data came from the acute or early subacute phase. Recordings within 24 hours to 10 days after stroke onset were reported in several cohorts using polysomnography (PSG) or cardiorespiratory monitoring [5, 6, 7, 8, 9, 11, 13, 14, 15]. Subacute or early rehabilitation phase CSA was described around 40–44 days after stroke in a rehabilitation unit cohort [10], and within approximately one week in another full‑night PSG study [16]. Chronic post‑stroke CSA or coexisting central sleep apnea was assessed ≥3 months after first ischemic stroke in a monocentric cohort of 185 patients [3], in patients treated with adaptive servo‑ventilation (ASV) a median of 11 months after stroke [18], and at 3–6 months in a longitudinal unilateral lateral medullary infarction series [13]. Study designs included prospective observational cohorts, case–control studies, single‑patient case reports and small case series, as well as narrative and systematic reviews that pooled central versus obstructive patterns but did not always provide separate CSA indices [19, 20]. Several studies enrolled mixed ischemic and hemorrhagic stroke populations, but where CSA indices were reported separately or ischemic stroke predominated, only the CSA‑related ischemic stroke data were considered [7, 12].

### 3.2 Definitions and Diagnostic Methods for CSA

Definitions and diagnostic modalities for CSA varied substantially between studies. Many cohorts used full‑night attended PSG, including EEG and standard respiratory channels, to identify central versus obstructive events by the absence of thoraco‑abdominal effort [10, 12, 13, 16, 23, 44]. Central sleep apnea was commonly defined as CSA when ≥50% of respiratory events were central and the apnea–hypopnea index (AHI) exceeded a threshold (e.g., AHI ≥5 or ≥10 events per hour), with a central AHI (cAHI) or central apnea index (CAI) used to quantify central burden [10, 17, 18, 44].

CSR and central periodic breathing during sleep (CPBS) were usually defined by a crescendo–decrescendo pattern of tidal volume and central apneas/hypopneas. Kim *et al*. [11] defined CSR as ≥3 consecutive central apneas/hypopneas with a crescendo–decrescendo breathing pattern, cycle length ≥40 seconds, and ≥5 central events per hour recorded over at least 2 hours. Hermann *et al*. [8] and Siccoli *et al*. [9] defined CPBS/CPB as ≥3 cycles of crescendo–decrescendo breathing with ≥50% reduction in nasal airflow lasting ≥10 seconds, with central patterns confirmed by reduced respiratory effort. Some acute studies relied on limited‑channel cardiorespiratory polygraphy without EEG [7, 9, 14, 15], using nasal pressure, thoracic and abdominal bands, and pulse oximetry to classify central events and CPB/CSR, but did not always provide explicit CAI thresholds for CSA as a syndrome. The large ischemic stroke HSAT cohort used a home sleep apnea test and defined CSA as CAI ≥5 events per hour with ≥50% of respiratory events being central [17].

Several case reports and small series documented CSA conversion or REM‑predominant CSA after stroke using PSG or portable monitoring. De Paolis *et al*. [26] reported an increase in central apnea index from 3 to 56 events per hour three days after a cardioembolic ischemic stroke, with total AHI 70 events per hour. Qu *et al*. [22] reported REM‑predominant CSA after medullary infarction with a baseline central AHI of 70.1 events per hour. Older acute ischemic stroke series assessed CSR using bedside respiratory motion and pulse oximetry without EEG or detailed CAI; Nachtmann *et al*. [5] defined CSR as periodic modulation of respiratory amplitude >50% of depth occurring more than 10 times per hour, without a numeric central index.

In several reviews [19, 20, 30, 31], CSA was conceptually defined as central apneas with absent respiratory effort and often as ≥50% central events within total AHI, but explicit numeric CAI cut‑offs specific to stroke cohorts were not consistently provided. A number of included stroke sleep‑disordered breathing studies focused on obstructive sleep apnea and total AHI without a separate central definition, and therefore did not contribute detailed CSA diagnostic criteria.

### 3.3 Prevalence and Severity of CSA Syndromes

In acute ischemic stroke cohorts, the proportion of patients with CSR ranged from approximately 19% to more than 50% when defined by pattern rather than CAI alone. In an acute ischemic stroke cohort of 182 patients assessed with overnight sleep apnea testing, CSR was present in 35 patients (19.1%) [11]. In a 156‑patient acute stroke cohort monitored within 24 hours of onset, CPB was present in 33 of 138 patients with usable breathing recordings (24%) when defined as central crescendo–decrescendo periodic breathing occupying a quantifiable fraction of recording time [7]. In a 32‑patient acute ischemic stroke series with bedside respiratory monitoring, CSR was detected in 17 of 32 patients (53%) [5].

When CSA was defined by cAHI or CAI thresholds, prevalence estimates tended to be lower. In a rehabilitation cohort of 93 stroke patients, CSA (central AHI ≥10 events per hour) was present in 19% [10]. In the ischemic stroke HSAT cohort (1346 patients), CSA (CAI ≥5 events per hour and ≥50% of events central) was found in 1.4% (19/1346) [17]. In the multinational SAS‑CARE 1 PSG cohort including mainly ischemic strokes and transient ischemic attacks, CSA prevalence was reported at 13–17%, with CSR present in 21–25% at baseline, decreasing at 3‑month follow‑up [12].

Central indices also showed broad ranges. In Siccoli *et al*. [9], the central apnea index in 74 acute first‑ever ischemic stroke patients averaged 7 ± 12 events per hour (range 0–61), and in those with sleep apnea (AHI >10 events per hour) the CAI averaged 12 ± 14 events per hour (range 0–61). In Martínez García *et al*. [6], among 139 acute ischemic stroke patients studied within 72 hours, the central apnea index was 2.4 ± 2.3 events per hour overall, with higher values in documented cerebrovascular accidents versus transient ischemic attacks (2.9 ± 2.2 vs 1.9 ± 2.2 events per hour) and in nocturnal strokes versus daytime strokes (3.2 ± 1.8 vs 1.7 ± 1.5 events per hour).

In lesion‑specific cohorts, central indices were often high. In 28 patients with unilateral lateral medullary infarction, median acute AHI was 16.5 (interquartile range 5.5–43.3), cAHI 11.0 (3.3–29.1), and CAI 8.3 (2.2–23.8) events per hour; CSA alone was present in 43% and mixed patterns in additional patients [13]. In Riglietti *et al*. [15], dominant CSA (≥70% of events central and central AHI ≥5 events per hour) in an acute cardiovascular–autonomic stroke cohort had mean central AHI 27.5 ± 15.1 events per hour, with central AHI across the cohort decreasing from 9.4 ± 12 to 4.0 ± 5.3 events per hour over 3 months.

Case‑level CSA severity was very high in some reports. In two fatal CSA cases with medullary lesions, 38 and 35 central apneas per hour were documented [21], and REM‑predominant CSA after bulbar infarction showed central AHI 70.1 events per hour [22]. In a case of CSA conversion after stroke, central apnea index increased from 3 to 56 events per hour three days after a cardioembolic ischemic stroke, with total AHI 70 events per hour [26].

One meta‑analysis that included mixed stroke types reported CSA prevalence 10.0% (95% confidence interval 6.5–14.9%) among stroke patients when CSA was defined as ≥50% central events within AHI, while obstructive events accounted for a higher proportion [20]. That pooled estimate did not provide ischemic stroke‑only prevalence and combined different phases and etiologies.

### 3.4 Clinical Correlates and Comorbidities

Cardiac comorbidities were frequently evaluated. In Nopmaneejumruslers *et al*. [10], CSA (central AHI ≥10 events per hour) was associated with lower mean nocturnal transcutaneous carbon dioxide tension (39.5 vs 43.0 mmHg) and a higher prevalence of left ventricular ejection fraction (LVEF) ≤40% (22% vs 5%) compared with non‑CSA stroke patients. In the 182‑patient acute ischemic stroke cohort [11], CSR patients were about nine years older on average and had higher frequencies of atrial fibrillation (31.4% vs 9.5%) and heart failure (8.6% vs 0.0%) than non‑CSR patients, as well as larger left atrial size and left‑atrial volume index and a higher proportion with LVEF <50%.

In multivariable logistic regression, Kim *et al*. [11] found that previous modified Rankin Scale (mRS) score, bilateral hemispheric lesions, left‑atrial volume index (per 10 mL/m^2^) and LVEF <50% remained independently associated with CSR, whereas age, hypertension, and smoking were not significant after adjustment. CSR was uncommon in small‑vessel occlusion strokes (2.86%) compared with other Trial of Org 10172 in Acute Stroke Treatment (TOAST) subtypes in that study.

Rowat *et al*. [7] reported that CPB presence was associated with more severe strokes (higher National Institutes of Health Stroke Scale, NIHSS), congestive heart failure, dysphagia, and reduced consciousness, while severity of prior cerebrovascular disease was not associated with CPB. Siccoli *et al*. [9] found that more severe CPBS was associated with older age, higher stroke severity, electrocardiographic abnormalities, and lower LVEF.

Age, sex and body mass index (BMI) were recurrent correlates of sleep‑disordered breathing overall, although not always specifically of CSA. In the chronic Baillieul cohort [44], moderate–severe sleep‑disordered breathing (SDB), including coexisting or central sleep apnea defined by cAHI/AHI ratios, was associated with male sex, obesity, age ≥65 years, and infratentorial lesions; coexisting/central sleep apnea had a higher frequency of cerebellar lesions and increased hypercapnic ventilatory response compared with no or mild SDB. In the ischemic stroke HSAT cohort, CSA cases (1.4% overall) were more often male and non‑obese, and CSA was not significantly associated with NIHSS, heart failure or most other comorbidities in that dataset [17].

Other vascular risk factors showed inconsistent relationships with CSA across studies. In Kim *et al*. [11], hypertension was more frequent in the CSA group in univariate analysis (71.4% vs 55.8%) but was not independently associated with CSA, and smoking was less frequent in the CSA group but did not remain significant after adjustment. In Nopmaneejumruslers *et al*. [10] and some other cohorts, stroke type and location were not significantly related to CSA, whereas cardiac function parameters were. Some physiological cohorts described CSA in relation to autonomic measures. Riglietti *et al*. [15] reported that CSA and mixed CSA/OSA patterns in acute stroke were associated with specific autonomic profiles (e.g., higher baroreflex gain and autonomic nervous system indexes) compared with obstructive patterns, but numerical autonomic values were study‑specific and not directly comparable with other cohorts. In an asymptomatic carotid stenosis cohort (not post‑stroke), CSA prevalence of 39% among patients with extracranial internal carotid artery stenosis was linked to severe stenosis and autonomic imbalance [27], but this population was not included in the ischemic stroke clinical outcome analyses.

### 3.5 Lesion Location and Neuroimaging Findings

In large acute hemispheric and mixed stroke cohorts, some studies did not find strong associations between CSA and specific vascular territories, while others highlighted bilateral or infratentorial involvement. In the study of Kim *et al*. [11], CSA presence was not significantly associated with vascular territory, supra‑ versus infratentorial location, or laterality when considered separately; however, bilateral hemispheric lesions were more frequent in the CSA group and remained independently associated with CSA in multivariable analysis. In Nachtmann *et al*. [5], CSA was present in 59% of supratentorial and 40% of infratentorial ischemic strokes, and the authors reported no clear dependence of CSA occurrence on infarct location when categorized simply as supratentorial versus infratentorial.

Martínez García *et al*. [6] reported that central apnea index and CSA were relatively uncommon (five patients with Cheyne–Stokes breathing) and that polysomnographic variables, including the central apnea index, did not vary significantly according to stroke site (left hemisphere, right hemisphere, vertebrobasilar territory, or undetermined) or lacunar versus non‑lacunar extent. Nocturnal‑onset strokes in that cohort, however, had higher total AHI and higher central apnea index than daytime strokes.

Some studies focused on specific lesion locations. Hermann *et al*. [8] described three patients with CPBS among 31 acute first‑ever stroke patients; lesions involved the left cingulate cortex, left insula, and right paramedian thalamus on diffusion‑weighted MRI, and CPBS occupied 18–24% of total sleep time, improving on follow‑up recording. Pavšič *et al*. [13] investigated 28 patients with unilateral lateral medullary infarction and found that CSA, defined by central AHI ≥5 events per hour with obstructive AHI <5 events per hour, and mixed CSA/OSA were very frequent in this brainstem infarction cohort; central indices decreased over 3–6 months.

Rowat *et al*. [7] reported that CPB was associated with large acute cerebral hemispheric lesions and severe mass effect on neuroimaging, though detailed volumetric data were not provided; CPB was not associated with the severity of prior cerebrovascular disease. Siccoli *et al*. [9] found that CPBS was more severe in extensive hemispheric strokes and less frequent in strokes involving the left insula and mesencephalon, based on imaging classifications. In the cohort of Baillieul *et al*. [44], coexisting or central sleep apnea among patients with moderate–severe SDB was associated with a higher frequency of cerebellar lesions, and moderate–severe SDB in general was associated with infratentorial lesions.

In the observational cohort of Kim *et al*. [11], a subgroup analysis limited to large‑artery atherosclerosis without cardiac disease showed that patients with CSA were older and more likely to have both supra‑ and infratentorial lesions, but in multivariable analysis only the previous mRS score remained significant in that subgroup. Several narrative reviews [30, 31, 40] discussed medullary, pontine, insular and thalamic involvement in relation to CSA, but did not contribute new, independent lesion–CSA datasets beyond the primary studies already described.

A substantial proportion of the 55 studies did not systematically relate CSA to detailed neuroimaging findings; in many of those studies, lesion location was either not reported in a form usable for CSA‑specific correlation or was described without stratifying respiratory patterns by location.

### 3.6 Clinical Outcomes (Functional Status, Complications, Mortality)

In Rowat *et al*. [7], CPB presence was independently associated with death or dependency (modified Rankin Scale ≥3) at 3 months; the adjusted odds ratio for this combined endpoint was approximately 5.9, and patients with CPB had higher mortality rates and lower Barthel Index scores than those without CPB. CPB was also associated with acute complications such as dysphagia and reduced consciousness.

In the SAS‑CARE 1 cohort [12], higher baseline AHI (including central and obstructive events) was associated with poor functional outcome (mRS ≥3) at 3 months, and the mean AHI decreased modestly between baseline and follow‑up; CSA proportions declined over this interval but CSA‑specific outcome analyses were not separated from overall AHI. Thus, a direct estimate of functional or mortality risk attributable exclusively to CSA was not reported.

In Mekky *et al*. [16], stroke patients had higher AHI, CSA index and desaturation index and lower minimal oxygen saturation compared with age‑matched controls; within the stroke group, lower minimal SpO_2_ at one week correlated with worse NIHSS at one month. The study did not report functional outcomes stratified solely by CSA status, and no mortality analysis was presented specific to CSA.

In Nopmaneejumruslers *et al*. [10], clinical outcomes such as mortality or long‑term disability by CSA status were not the primary focus; the study concentrated on CSA prevalence and its relationship with cardiac function and transcutaneous carbon dioxide tension (CSA‑specific outcomes not reported). In Pavšič *et al*. [13], outcomes of interest were changes in AHI and cAHI over time in unilateral lateral medullary infarction; functional or mortality endpoints stratified by CSA were not detailed (not reported).

Kim *et al*. [11] reported baseline NIHSS and premorbid mRS differences between CSR and non‑CSR groups but did not present separate long‑term functional outcome or mortality rates according to CSR status beyond the logistic regression models for CSR determinants (CSR‑specific prognostic data not reported). In Martínez García *et al*. [6] and Nachtmann *et al*. [5], respiratory patterns were described and comparisons with general population or control data were made, but CSA‑specific mortality or disability statistics were not reported.

Narrative reviews and meta‑analyses [19, 20, 31, 39, 40] commonly stated that CSA after stroke are associated with more severe neurological impairment or higher mortality, but did not provide new numerical outcome data for CSA‑only groups beyond the primary studies already summarized.

Therapeutic data were limited to small observational reports and case series; one retrospective series found that adaptive servoventilation reduced apnea–hypopnea index and improved symptoms in post-acute stroke patients with persistent CSA, while other reports described partial spontaneous resolution or treatment-related changes in central indices [18, 22, 26].

Overall, among the 55 studies included in this scoping review, a limited subset provided quantitative outcome measures that were explicitly stratified by CSA, CPB or CSR status in ischemic stroke; in many cohorts, outcomes were reported for sleep‑disordered breathing as a whole or were not separated by respiratory pattern, resulting in “not reported” for CSA‑specific functional status, complication rates or mortality in a considerable proportion of the evidence base.

## 4. Discussion

Across the 55 studies, CSA appear as context‑dependent phenomena rather than a single, uniform syndrome after ischemic stroke. High CSA rates in acute, pattern‑based cohorts contrast with low CSA prevalence when strict central apnea index thresholds or home testing are used, showing that prevalence largely reflects how “central” breathing is defined and measured [5, 7, 10, 11, 55]. The consistent clustering of central patterns in patients with cardiac dysfunction, prior disability and more extensive or bilateral lesions suggests that CSA after stroke usually marks combined cardio‑cerebrovascular vulnerability rather than an isolated respiratory problem [7, 9, 10, 11, 44].

### 4.1 Pathophysiological Considerations

The variability in CSA prevalence and phenotype can be explained by the different ways stroke and comorbid disease alter ventilatory control. Unilateral lesions in insula, cingulate cortex, thalamus and medulla were sufficient to generate CPB or CSA in patients without severe heart disease, with partial resolution over weeks or months, supporting a direct neurogenic contribution from damage to central respiratory networks [8, 13, 21]. At the same time, several cohorts showed that central patterns concentrate in patients with reduced left ventricular ejection fraction, enlarged left atrium, lower nocturnal carbon dioxide and atrial fibrillation, indicating that circulation delay and high loop gain frequently interact with stroke‑related injury to produce CSA [10, 11]. Longitudinal data—central indices falling in some lesion‑focused series but rising in broader stroke cohorts—suggest that as the brain recovers and cardiac status evolves, the balance between obstructive and central events shifts, which accounts for apparently conflicting time‑course findings [8, 12, 13, 15, 57].

The pathophysiology of CSA in ischemic stroke appears multifactorial, involving disruption of central respiratory networks and interaction with cardiac dysfunction and ventilatory instability. From a clinical standpoint, identifying CSA may help risk-stratify stroke patients, but the evidence for specific management strategies remains limited and largely non-randomized.

### 4.2 Clinical Implications for Stroke Care

**Fig. 2.**
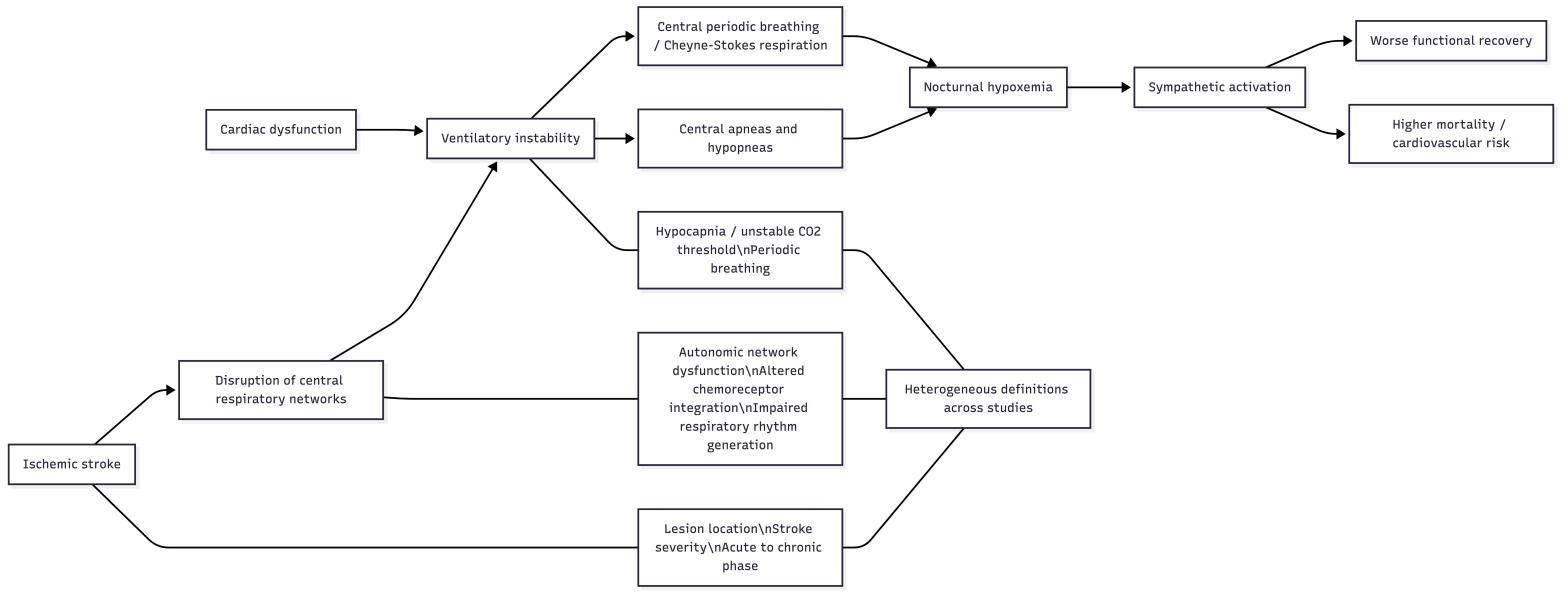
**Conceptual framework of central sleep apnea after ischemic stroke**. The figure summarizes the main pathways described in the literature, including disruption of central respiratory networks, altered chemoreceptive and autonomic control, ventilatory instability, and the interaction with cardiac dysfunction, which may lead to central apneas, periodic breathing, and adverse clinical outcomes.

These mechanisms help interpret why central patterns carry prognostic information in several studies. Because CSA tends to occur in patients with more severe neurological deficits and worse cardiac status, their presence identifies individuals who already have a high risk profile, which explains the observed associations with death or dependency rather than implying direct causality [7, 12, 23]. Early sleep testing captures transient but prognostically relevant central instabilities, yet feasibility constraints mean that the sickest patients are often not studied, so current data likely underestimate the burden of CSA in real‑world stroke [11, 58]. Case reports and small series showing conversion from obstructive to central patterns and response to bilevel or adaptive servo‑ventilation illustrate that management may need to adapt dynamically as central instability emerges, but also highlight that some central patterns resolve spontaneously, supporting careful, individualized decision‑making [18, 22, 26] (Fig. 2).

### 4.3 Knowledge Gaps and Future Research Directions

The way the existing evidence is structured points directly to what is needed next. Harmonized, stroke‑specific definitions that integrate central indices and pattern descriptors would reduce artificial heterogeneity and make future studies comparable [9, 10, 11, 44, 55]. Prospective multicenter cohorts that include severe strokes and repeat standardized sleep studies from the acute phase into the chronic stage are required to disentangle transient neurogenic CSA from more persistent, heart‑failure–related phenotypes and to clarify how lesion topography, cardiac function and autonomic markers jointly shape central instability [8, 12, 13, 15, 57]. Finally, pragmatic interventional trials embedded in stroke services—comparing diagnostic strategies and treatment pathways for patients with prominent central patterns—are needed to test whether identifying and treating CSA after ischemic stroke can meaningfully change functional outcomes or vascular risk, rather than simply describe another marker of severity [7, 18, 22, 26].

The current evidence supports a prioritized research agenda. Future studies should first adopt standardized, stroke-specific definitions of CSA and related central breathing phenotypes. They should then incorporate longitudinal phenotyping across the acute-to-chronic stroke continuum, stratify results by stroke subtype and lesion location, and evaluate potential modifiers such as sex, age, and cardiac function. Finally, pragmatic interventional studies are needed to determine whether identification and treatment of CSA after ischemic stroke improve functional recovery and other clinical outcomes.

#### 4.3.1 Limitations 

This scoping review has several limitations. It is based solely on the 55 available studies, which are heterogeneous in design, CSA definitions, sleep‑study methods, stroke phases and outcome reporting, and many cohorts exclude the most severe strokes or lack detailed CSA‑specific analyses, so selection and reporting biases cannot be excluded. These constraints limit the comparability of results and the strength of causal inferences regarding central sleep apnea after ischemic stroke.

#### 4.3.2 Knowledge Gaps 

Key knowledge gaps remain despite the 55 included studies. The absence of a harmonized, stroke‑specific definition of central sleep apnea (CSA) and Cheyne–Stokes respiration (CSR) means that current discrepancies in prevalence and risk estimates may reflect methodology more than biology [59, 60, 61]. Longitudinal information is limited to a few cohorts and does not clearly separate transient, stroke‑triggered central instability from more chronic, heart‑failure–driven CSA phenotypes. Lesion–symptom relationships and ischemic stroke subtypes are underexplored: small series suggest roles for insular, thalamic, cerebellar and medullary lesions, but larger imaging‑based cohorts stratified by lacunar, cardioembolic, atherothrombotic and other etiologies are lacking. Virtually no study systematically examines sex differences or focuses on very old patients (≥85 years), so potential modifiers by sex and advanced age remain unknown. Prognostic analyses rarely isolate the independent contribution of central events after accounting for obstructive indices and global severity, and interventional evidence is restricted to small, uncontrolled reports of bilevel or adaptive servo‑ventilation without randomized evaluation in post‑ischemic stroke populations.

## 5. Conclusions

Central sleep apnea and Cheyne–Stokes respiration after ischemic stroke emerge from the combined impact of focal brain lesions, cardiac dysfunction and timedependent changes in ventilatory control, clustering particularly in patients with bilateral or extensive lesions, reduced ejection fraction, atrial enlargement and higher premorbid disability. Existing studies suggest that these central patterns often signal a highrisk cardiocerebrovascular profile and may accompany poorer functional outcomes, but inconsistent definitions, heterogeneous methods, selective sampling and the very limited, smallscale experience with adaptive servoventilation or other targeted therapies—without any randomized trials—mean that their independent prognostic weight and treatability remain uncertain, underscoring the need for standardized criteria, broader longitudinal cohorts and robust intervention studies specifically evaluating servoventilation and related approaches in this population.

## Availability of Data and Materials

The datasets generated and analyzed during this scoping review are available from the corresponding author on reasonable request.

## Supplementary Material

Supplementary material associated with this article can be found, in the online version, at https://doi.org/10.31083/RN49726.
