# Supplementary material for: Mapping the Evidence: Central Sleep Apnea Syndromes During Sleep and Stroke—A Scoping Review
Source: Rev Neurol. 2026 May 26;81(5):49726. doi: 10.31083/RN49726 (PMC13221676; doi:10.31083/RN49726)
Supplement: Supplementary file 1 [file 1576-6578-81-5-49726-s1.zip › Supplementary Material-I.docx]

**Complete search strategy**

**PubMed**

((("ischemic stroke"[MeSH Terms] OR ("ischemic"[All Fields] AND "stroke"[All Fields]) OR "ischemic stroke"[All Fields] OR ("brain stem infarctions"[MeSH Terms] OR ("brain"[All Fields] AND "stem"[All Fields] AND "infarctions"[All Fields]) OR "brain stem infarctions"[All Fields] OR ("brain"[All Fields] AND "stem"[All Fields] AND "infarction"[All Fields]) OR "brain stem infarction"[All Fields]) OR ("cerebral infarction"[MeSH Terms] OR ("cerebral"[All Fields] AND "infarction"[All Fields]) OR "cerebral infarction"[All Fields]) OR ("brain ischaemia"[All Fields] OR "brain ischemia"[MeSH Terms] OR ("brain"[All Fields] AND "ischemia"[All Fields]) OR "brain ischemia"[All Fields]) OR ("cerebrovascular disorders"[MeSH Terms] OR ("cerebrovascular"[All Fields] AND "disorders"[All Fields]) OR "cerebrovascular disorders"[All Fields]))

AND

("sleep apnea, central"[MeSH Terms] OR ("sleep"[All Fields] AND "apnea"[All Fields] AND "central"[All Fields]) OR "central sleep apnea"[All Fields] OR "sleep apnea central"[All Fields] OR (("period"[All Fields] OR "periodic"[All Fields] OR "periodical"[All Fields] OR "periodically"[All Fields] OR "periodicals"[All Fields] OR "periodicity"[MeSH Terms] OR "periodicity"[All Fields] OR "periodicities"[All Fields] OR "periods"[All Fields]) AND ("breath"[All Fields] OR "breathe"[All Fields] OR "breathed"[All Fields] OR "breathes"[All Fields] OR "breathings"[All Fields] OR "breaths"[All Fields] OR "respiration"[MeSH Terms] OR "respiration"[All Fields] OR "breathing"[All Fields])) OR ("cheyne stokes respiration"[MeSH Terms] OR ("cheyne stokes"[All Fields] AND "respiration"[All Fields]) OR "cheyne stokes respiration"[All Fields] OR ("cheyne"[All Fields] AND "stokes"[All Fields] AND "respiration"[All Fields]) OR "cheyne stokes respiration"[All Fields]) OR (("central"[All Fields] OR "centrally"[All Fields] OR "centrals"[All Fields]) AND ("breath"[All Fields] OR "breathe"[All Fields] OR "breathed"[All Fields] OR "breathes"[All Fields] OR "breathings"[All Fields] OR "breaths"[All Fields] OR "respiration"[MeSH Terms] OR "respiration"[All Fields] OR "breathing"[All Fields]) AND ("disease"[MeSH Terms] OR "disease"[All Fields] OR "disorder"[All Fields] OR "disorders"[All Fields] OR "disorder s"[All Fields] OR "disordes"[All Fields])))

AND

("english"[Language] OR "spanish"[Language]))

NOT

("child*"[All Fields] OR ("paediatrics"[All Fields] OR "pediatrics"[MeSH Terms] OR "pediatrics"[All Fields] OR "paediatric"[All Fields] OR "pediatric"[All Fields])))

| **Translations**  **ischemic stroke:** "ischemic stroke"[MeSH Terms] OR ("ischemic"[All Fields] AND "stroke"[All Fields]) OR "ischemic stroke"[All Fields]  **brain stem infarction:** "brain stem infarctions"[MeSH Terms] OR ("brain"[All Fields] AND "stem"[All Fields] AND "infarctions"[All Fields]) OR "brain stem infarctions"[All Fields] OR ("brain"[All Fields] AND "stem"[All Fields] AND "infarction"[All Fields]) OR "brain stem infarction"[All Fields]  **cerebral infarction:** "cerebral infarction"[MeSH Terms] OR ("cerebral"[All Fields] AND "infarction"[All Fields]) OR "cerebral infarction"[All Fields]  **brain ischemia:** "brain ischaemia"[All Fields] OR "brain ischemia"[MeSH Terms] OR ("brain"[All Fields] AND "ischemia"[All Fields]) OR "brain ischemia"[All Fields]  **cerebrovascular disorders:** "cerebrovascular disorders"[MeSH Terms] OR ("cerebrovascular"[All Fields] AND "disorders"[All Fields]) OR "cerebrovascular disorders"[All Fields]  **sleep apnea, central:** "sleep apnea, central"[MeSH Terms] OR ("sleep"[All Fields] AND "apnea"[All Fields] AND "central"[All Fields]) OR "central sleep apnea"[All Fields] OR "sleep apnea, central"[All Fields]  **periodic:** "period"[All Fields] OR "periodic"[All Fields] OR "periodical"[All Fields] OR "periodically"[All Fields] OR "periodicals"[All Fields] OR "periodicity"[MeSH Terms] OR "periodicity"[All Fields] OR "periodicities"[All Fields] OR "periods"[All Fields]  **breathing:** "breath"[All Fields] OR "breathe"[All Fields] OR "breathed"[All Fields] OR "breathes"[All Fields] OR "breathings"[All Fields] OR "breaths"[All Fields] OR "respiration"[MeSH Terms] OR "respiration"[All Fields] OR "breathing"[All Fields]  **cheyne-stokes respiration:** "cheyne-stokes respiration"[MeSH Terms] OR ("cheyne-stokes"[All Fields] AND "respiration"[All Fields]) OR "cheyne-stokes respiration"[All Fields] OR ("cheyne"[All Fields] AND "stokes"[All Fields] AND "respiration"[All Fields]) OR "cheyne stokes respiration"[All Fields]  **central:** "central"[All Fields] OR "centrally"[All Fields] OR "centrals"[All Fields]  **breathing:** "breath"[All Fields] OR "breathe"[All Fields] OR "breathed"[All Fields] OR "breathes"[All Fields] OR "breathings"[All Fields] OR "breaths"[All Fields] OR "respiration"[MeSH Terms] OR "respiration"[All Fields] OR "breathing"[All Fields]  **disorders:** "disease"[MeSH Terms] OR "disease"[All Fields] OR "disorder"[All Fields] OR "disorders"[All Fields] OR "disorder's"[All Fields] OR "disordes"[All Fields]  **english[Filter]:** english [LA]  **spanish[Filter]:** spanish [LA]  **pediatric:** "paediatrics"[All Fields] OR "pediatrics"[MeSH Terms] OR "pediatrics"[All Fields] OR "paediatric"[All Fields] OR "pediatric"[All Fields] |  |
| --- | --- |

636 Documents

**SCOPUS**

( TITLE-ABS-KEY ( "ischemic stroke" OR "brain stem infarction" OR "cerebral infarction" OR "brain ischemia" OR "cerebrovascular disorders" )

AND

TITLE-ABS-KEY ( "central sleep apnea" OR "periodic breathing" OR "cheyne-stokes" OR "central apnea" )

AND NOT

TITLE-ABS-KEY ( child* OR pediatric ) )

AND

( LIMIT-TO ( LANGUAGE , "English" ) OR LIMIT-TO ( LANGUAGE , "Spanish" ) )

109 Documents

**Web of Science Search Strategy** (v0.1)

# Database: Web of Science Core Collection
# Entitlements:
- WOS.IC: 1993 to 2025
- WOS.CCR: 1985 to 2025
- WOS.SCI: 1900 to 2025
- WOS.AHCI: 1975 to 2025
- WOS.BHCI: 2005 to 2025
- WOS.BSCI: 2005 to 2025
- WOS.ESCI: 2020 to 2025
- WOS.ISTP: 1990 to 2025
- WOS.SSCI: 1956 to 2025
- WOS.ISSHP: 1990 to 2025

# Searches:

ischemic stroke OR brain stem infarction OR cerebral infarction OR brain ischemia OR cerebrovascular disorders (All Fields)

AND

central sleep apnea OR periodic breathing OR cheyne-stokes respiration OR central breathing disorders (All Fields)

NOT

child* OR pediatric (All Fields)

AND

Spanish or English (Languages)

Date Run: Wed Aug 31 2025 14:34:29 GMT+0200 (hora de verano de Europa central)

Results: 239

**Cochrane**

ID Search Hits

#1 ischemic stroke OR brain stem infarction OR cerebral infarction OR brain ischemia OR cerebrovascular disorders 33344

#2 sleep apnea, central OR periodic breathing OR cheyne-stokes respiration OR central breathing disorders 12498

#3 #1 AND #2 350

#4 child* OR pediatric 256992

#5 #3 NOT #4

Results: **280**
