# Supplementary material for: Mapping the Evidence: Central Sleep Apnea Syndromes During Sleep and Stroke—A Scoping Review
Source: Rev Neurol. 2026 May 26;81(5):49726. doi: 10.31083/RN49726 (PMC13221676; doi:10.31083/RN49726)
Supplement: Supplementary file 1 [file 1576-6578-81-5-49726-s1.zip › Supplementary Material-II.docx]

|  |  | **Eligibility** | **Comments** |
| --- | --- | --- | --- |
| Reference |  | **Yes/ No / Unclear** | (Bias, conflicts of interests, etc.) |
| Author & Year, Country (where the study was conducted) |  |  |  |
| Study design |  |  |  |
| Sample size (n)  (population, sex, average age subjects, year + SD) |  |  |  |
| Stroke phase   - Acute - Subacute - Chronic |  |  |  |
| Stroke type   - cardioembolic stroke - lacunar infarct - infarct of unusual etiology - essential cerebral infarct, - atherothrombotic infarct, - intracerebral hemorrhage |  |  |  |
| Lesion location |  |  |  |
| Diagnostic methods |  |  |  |
| Quantitative respiratory indices  (CAI, Cheyne–Stokes respiration percentage, total AHI) |  |  |  |
| Comorbidities  (atrial fibrillation, congestive heart failure, pulmonary disease) |  |  |  |
| Reported outcomes  including   - neurological severity (NIHSS), - functional recovery (mRS, Barthel Index), - mortality, - therapeutic interventions (e.g., CPAP or adaptive servo‑ventilation) |  |  |  |
| Level of evidence |  |  |  |
