# Supplementary material for: Mapping the Evidence: Central Sleep Apnea Syndromes During Sleep and Stroke—A Scoping Review
Source: Rev Neurol. 2026 May 26;81(5):49726. doi: 10.31083/RN49726 (PMC13221676; doi:10.31083/RN49726)
Supplement: Supplementary file 1 [file 1576-6578-81-5-49726-s1.zip › Supplementary Material-PRISMA checklist.docx]

PRISMA 2020 Checklist

*"Mapping the Evidence: Central Sleep Apnea Syndromes During Sleep and Stroke—A Scoping Review"*

| **Section and Topic** | **Item #** | **Checklist item** | **Location where item is reported** |
| --- | --- | --- | --- |
| **TITLE** |  |  |  |
| **Title** | 1 | Identify the report as a systematic review. | Title page: "Mapping the Evidence: Central Sleep Apnea Syndromes During Sleep and Stroke---A Scoping Review" |
| **ABSTRACT** |  |  |  |
| **Abstract** | 2 | See the PRISMA 2020 for Abstracts checklist. | Abstract section (p. 1): Includes background, methods, results, and conclusions structured according to PRISMA format with research questions, databases searched, inclusion criteria, number of studies reviewed (55), prevalence findings (8-12%), key pathophysiological insights, and conclusions regarding heterogeneity limitations |
| **INTRODUCTION** |  |  |  |
| **Rationale** | 3 | Describe the rationale for the review in the context of existing knowledge. | Introduction section (pp. 1-2): Establishes that CSA is underexplored compared to OSA in stroke; CSA prevalence substantially elevated in stroke cohorts (8-12%) vs. general population (<1%); explains clinical relevance of understanding CSA mechanisms and outcomes in cerebrovascular disease; notes gaps in current evidence-based management strategies |
| **Objectives** | 4 | Provide an explicit statement of the objective(s) or question(s) the review addresses. | Materials and Methods section - "Eligibility criteria" (p. 3): Primary research question explicitly stated: "What is the extent of evidence on the prevalence, clinical manifestations, diagnostic methods, management strategies, and outcomes of CSA syndromes in adults with ischemic stroke across different care settings and disease phases?" |
| **METHODS** |  |  |  |
| **Eligibility criteria** | 5 | Specify the inclusion and exclusion criteria for the review and how studies were grouped for the syntheses. | Materials and Methods - "Eligibility criteria" (p. 3): Population defined as adults ≥18 years with ischemic stroke confirmed by neuroimaging and CAI ≥5 events/hour; Concept: CSA syndromes impact on stroke patients; Context: any healthcare setting and any disease phase (acute <7 days, subacute 1-3 months, chronic >3 months). Exclusion criteria explicitly listed: pediatric/non-human populations, editorials/commentaries, conference abstracts without full text, non-English/Spanish publications, papers without specific CSA data or confirmed ischemic stroke populations |
| **Information sources** | 6 | Specify all databases, registers, websites, organisations, reference lists and other sources searched or consulted to identify studies. Specify the date when each source was last searched or consulted. | Materials and Methods - "Information sources" (p. 3): Four databases searched: PubMed, Scopus, Web of Science (Core Collection), and Cochrane Library from inception to 31 August 2025. Searches restricted to English and Spanish language publications. Reference lists of all included papers and relevant systematic reviews manually examined for additional studies. Grey literature and non-peer-reviewed publications excluded |
| **Search strategy** | 7 | Present the full search strategies for all databases, registers and websites, including any filters and limits used. | Materials and Methods - "Information sources" (p. 3): Initial pilot search performed in PubMed to identify MeSH terms and free-text terms organized in two broad blocks: (1) "sleep apnea, central" ("periodic breathing", "Cheyne-Stokes respiration" or "central breathing disorders") and (2) ischemic stroke ("brain stem infarctions" or "cerebral infarction" or "brain infarction" or "brain ischemia" or "cerebrovascular disorders"). Final PubMed strategy adapted for other databases. Full search strategy for all databases provided in Supplementary Material I |
| **Selection process** | 8 | Specify the methods used to decide whether a study met the inclusion criteria of the review, including how many reviewers screened each record and each report retrieved, whether they worked independently, and if applicable, details of automation tools used in the process. | Materials and Methods - "Study selection and data extraction" (p. 3-4): Two independent reviewers screened article titles and abstracts using predetermined inclusion criteria in first stage. Second stage: full-text versions evaluated independently by same two reviewers. Disagreements resolved by discussion or consultation with third reviewer. References imported into Zotero 7 for Windows (v. 7.0.22) for reference management and duplicate removal |
| **Data collection process** | 9 | Specify the methods used to collect data from reports, including how many reviewers collected data from each report, whether they worked independently, any processes for obtaining or confirming data from study investigators, and if applicable, details of automation tools used in the process. | Materials and Methods - "Study selection and data extraction" (pp. 3-4): Data extraction conducted independently by two reviewers using customized Microsoft Excel-based extraction form designed specifically for review (Supplementary Material II). Inconsistencies identified during data extraction discussed and resolved by consensus. Revisions to extraction form documented to ensure transparency |
| **Data items** | 10a | List and define all outcomes for which data were sought. Specify whether all results that were compatible with each outcome domain in each study were sought (e.g. for all measures, time points, analyses), and if not, the methods used to decide which results to collect. | Materials and Methods - "Data items" (p. 4): Outcomes extracted included: neurological severity (NIHSS), functional recovery (modified Rankin Scale/mRS, Barthel Index), mortality, and cardiovascular outcomes. Diagnostic indices extracted: central apnea index (CAI), Cheyne-Stokes respiration percentage, total apnea-hypopnea index (AHI) |
|  | 10b | List and define all other variables for which data were sought (e.g. participant and intervention characteristics, funding sources). Describe any assumptions made about any missing or unclear information. | Materials and Methods - "Data items" (p. 4): Variables extracted organized as follows: study characteristics (design, country, publication year); population data and stroke characteristics (phase: acute/subacute/chronic, lesion location); diagnostic procedures applied for identifying CSA syndromes; main clinical and neurological outcomes (NIHSS, mRS, mortality); reported management and therapeutic strategies for CSA syndromes (CPAP, ASV, non-invasive ventilation). Additionally extracted: authorship, year, sample size, stroke type, phase of disease, comorbidities (atrial fibrillation, congestive heart failure, pulmonary disease), therapeutic interventions. Each article's main findings summarized to describe scope and nature of evidence |
| **Study risk of bias assessment** | 11 | Specify the methods used to assess risk of bias in the included studies, including details of the tool(s) used, how many reviewers assessed each study and whether they worked independently, and if applicable, details of automation tools used in the process. | Materials and Methods: Not explicitly described in detail. Risk of bias assessed implicitly through level of evidence classification (OCEBM criteria) reported in Table 1, with studies stratified by design (Level 1b prospective cohorts, Level 2b/2 prospective observational studies, Level 4 case reports). No formal risk of bias tool (e.g., Cochrane RoB, ROBINS-I) mentioned; heterogeneity acknowledged as limitation |
| **Effect measures** | 12 | Specify for each outcome the effect measure(s) (e.g. risk ratio, mean difference) used in the synthesis or presentation of results. | Materials and Methods - "Data synthesis" (p. 4): "Because of the methodological heterogeneity of included studies, a quantitative meta-analysis was not feasible." Instead, findings synthesized in narrative framework and systematically analyzed according to level of evidence following hierarchical standards of evidence-based medicine. No specific effect measures (e.g., RR, OR, MD) used given non-quantitative synthesis approach |
| **Synthesis methods** | 13a | Describe the processes used to decide which studies were eligible for each synthesis (e.g. tabulating the study intervention characteristics and comparing against the planned groups for each synthesis (item #5)). | Materials and Methods - "Data synthesis" (p. 4): Narrative synthesis approach. Studies grouped by: stroke phase (acute/subacute/chronic), diagnostic methods (polysomnography vs. portable polygraphy vs. home sleep testing), CSA definitions used, clinical outcomes reported. Results section organized by study design and phase of disease assessment |
|  | 13b | Describe any methods required to prepare the data for presentation or synthesis, such as handling of missing summary statistics, or data conversions. | Materials and Methods - "Data items" (p. 4): Missing or unclear information handled through consensus discussion by reviewers. Data standardization attempted where possible (e.g., CAI definitions, AHI reporting), though heterogeneity in reporting acknowledged as major limitation |
|  | 13c | Describe any methods used to tabulate or visually display results of individual studies and syntheses. | Results section: Table 1 presents comprehensive characteristics of 55 included studies organized by: author/year, study design, sample size, stroke phase, type of sleep study, CSA definition, CAI/AHI values, main clinical outcomes, and level of evidence. Figure 1 presents PRISMA 2020 Flow Diagram showing study selection process (1,264 records identified → 55 studies included) |
|  | 13d | Describe any methods used to synthesize results and provide a rationale for the choice(s). If meta-analysis was performed, describe the model(s), method(s) to identify the presence and extent of statistical heterogeneity, and software package(s) used. | Materials and Methods - "Data synthesis" (p. 4): Narrative synthesis chosen due to methodological heterogeneity of included studies. No meta-analysis performed. Findings synthesized in narrative framework and systematically analyzed according to level of evidence using hierarchical standards of evidence-based medicine. Rationale: heterogeneous CSA definitions, diagnostic protocols, study designs, and outcome measures precluded quantitative synthesis |
|  | 13e | Describe any methods used to explore possible causes of heterogeneity among study results (e.g., subgroup analysis, meta-regression). | Results and Discussion sections: Heterogeneity analyzed by: CSA definition used (CAI ≥5, ≥20, ≥50 events/hour; pattern-based CSR definitions), diagnostic method (polysomnography vs. portable devices vs. home sleep testing), stroke phase (acute vs. subacute vs. chronic), lesion location (supratentorial vs. infratentorial vs. cerebellar). Discussion addresses sources of heterogeneity and their impact on interpretability |
|  | 13f | Describe any sensitivity analyses conducted to assess robustness of the synthesized results. | Materials and Methods and Discussion: Explicit sensitivity analyses not reported. However, discussion acknowledges heterogeneity limitations and notes that conclusions differ based on CSA definition applied (8-12% with strict CAI cutoffs vs. 19-53% with pattern-based definitions). Discussion emphasizes need for standardized criteria |
| **Reporting bias assessment** | 14 | Describe any methods used to assess risk of bias due to missing results in a synthesis (arising from reporting biases). | Not explicitly addressed in methods. Discussion acknowledges: selective sampling (most cohorts exclude severe strokes), publication bias potential not formally assessed, gray literature excluded, non-peer-reviewed publications excluded |
| **Certainty assessment** | 15 | Describe any methods used to assess certainty (or confidence) in the body of evidence for an outcome. | Results - Table 1 (pp. 6-23): Level of Evidence assessed using OCEBM (Oxford Centre for Evidence-Based Medicine) criteria, stratified by study design: Level 1b (prospective cohorts), Level 2b (prospective observational), Level 2 (prospective observational with imaging), Level 4 (case reports/small series). Discussion notes limitations in certainty across outcomes due to heterogeneous definitions and small sample sizes in intervention studies |
| **RESULTS** |  |  |  |
| **Study selection** | 16a | Describe the results of the search and selection process, from the number of records identified in the search to the number of studies included in the review, ideally using a flow diagram. | Results - "Study selection and general characteristics" (p. 5): 1,264 records identified across databases (PubMed n=636, Scopus n=109, Web of Science Core Collection n=239, Cochrane n=280); 220 duplicate records and 2 additional records removed before screening; 1,042 records screened at title/abstract level; 712 excluded; 330 reports sought for retrieval; 62 could not be retrieved; 268 full texts assessed for eligibility; 213 excluded (study typology n=128, methodology n=38, population n=22, other reasons n=25); final inclusion of 55 studies. Figure 1 (p. 4): PRISMA 2020 Flow Diagram illustrates complete selection process |
|  | 16b | Cite studies that might appear to meet the inclusion criteria, but which were excluded, and explain why they were excluded. | Results - "Study selection and general characteristics" (p. 5): Exclusion reasons quantified: study typology (n=128 studies), methodology (n=38), population (n=22), other reasons (n=25). Specific examples: studies involving pediatric/non-human populations excluded; editorials, commentaries excluded; conference abstracts without full text excluded; non-English/Spanish publications excluded; papers without specific CSA data or without confirmed ischemic stroke populations excluded. Discussion notes several systematic reviews pooled central versus obstructive patterns without providing separate CSA indices and were excluded from quantitative synthesis |
| **Study characteristics** | 17 | Cite each included study and present its characteristics. | Results - Table 1 (pp. 6-23): Comprehensive presentation of all 55 included studies with characteristics including: author and year, study design, sample size, stroke phase, type of sleep study, CSA definition/index, CAI or AHI values, main clinical outcomes reported, and OCEBM level of evidence. Each row represents one included study with complete bibliographic and methodological information |
| **Risk of bias in studies** | 18 | Present assessments of risk of bias for each included study. | Results - Table 1 (pp. 6-23): Risk of bias assessed indirectly through OCEBM level of evidence (Level 1b through Level 4). Higher-level studies (Level 1b-2b) generally prospective cohorts; lower-level studies (Level 4) case reports/series. Discussion acknowledges limitations including: selective sampling (severe strokes excluded), heterogeneous definitions affecting validity, small sample sizes in intervention studies, incomplete lesion-CSA correlation analyses. Specific quality issues noted for individual studies (e.g., some failed to demonstrate consistent lesion-CSA associations) |
| **Results of individual studies** | 19 | For all outcomes, present, for each study: (a) summary statistics for each group (where appropriate) and (b) an effect estimate and its precision (e.g. confidence/credible interval), ideally using structured tables or plots. | Results - Table 1 (pp. 6-23): For each study, main summary statistics presented including: sample size, CSA prevalence (e.g., "42.9% among moderate-severe SDB"), CAI/AHI indices (e.g., "Central AHI reported [events/h]"), and clinical outcomes (e.g., "association with severity of prior cerebrovascular disease"). Results of individual studies presented narratively in text with specific findings cited with author and year |
| **Results of syntheses** | 20a | For each synthesis, briefly summarise the characteristics and risk of bias among contributing studies. | Results - "Study selection and general characteristics" section (p. 5) and Table 1 (pp. 6-23): Synthesis organized by study design and stroke phase. Most CSA data from acute/early subacute phase (within 24 hours to 10 days), with some subacute (40-44 days) and chronic (≥3 months) assessments. Study designs included prospective cohorts (20 studies with original CSA data), case-control, case series, systematic reviews. Characteristics and OCEBM levels presented for each included study |
|  | 20b | Present results of all statistical syntheses conducted. If meta-analysis was done, present for each the summary estimate and its precision (e.g. confidence/credible interval) and measures of statistical heterogeneity. If comparing groups, describe the direction of the effect. | Results section: No meta-analysis conducted due to methodological heterogeneity. Narrative synthesis of findings presented. Prevalence of post-stroke CSA summarized: approximately 8-12% using strict CAI definitions; 19-53% with pattern-based definitions; 1.4% with home sleep testing. Direction of associations: CSA associated with worse outcomes (higher mortality, poorer functional recovery, increased cardiovascular complications), more extensive/bilateral lesions, cardiac dysfunction |
|  | 20c | Present results of all investigations of possible causes of heterogeneity among study results. | Results and Discussion sections (pp. 4-27): Sources of heterogeneity identified and discussed: (1) CSA definition heterogeneity (CAI cutoffs ≥5, ≥20, ≥50 events/hour vs. pattern-based CSR definitions resulting in 1.4-53% prevalence estimates), (2) diagnostic method variation (polysomnography vs. portable devices vs. home sleep testing), (3) timing of assessment (acute vs. subacute vs. chronic), (4) lesion location inconsistency (some studies showed lesion-CSA correlation, others did not), (5) outcome measurement variation. Discussion emphasizes that "heterogeneity in CSA definitions, diagnostic protocols, and outcome measures limits firm conclusions" |
|  | 20d | Present results of all sensitivity analyses conducted to assess the robustness of the synthesized results. | Discussion section (pp. 26-27): Although formal sensitivity analyses not conducted, robustness considerations addressed: heterogeneous CSA definitions yielded dramatically different prevalence estimates (1.4% vs. 53%), suggesting findings sensitive to definition choice. Discussion notes conclusions vary substantially depending on whether strict CAI thresholds or pattern-based definitions applied, indicating methodological choices substantially impact findings |
| **Reporting biases** | 21 | Present assessments of risk of bias due to missing results (arising from reporting biases) for each synthesis assessed. | Discussion section (pp. 26-27): Publication bias not formally assessed. Acknowledged limitations: selective sampling in cohorts (most exclude severe strokes), exclusion of gray literature and non-peer-reviewed publications, potential for reporting bias in intervention studies (only safety and symptom improvement reported for CPAP/ASV, no randomized trials on stroke recurrence outcomes). Several studies noted to not show consistent lesion-CSA correlations, but negative results not systematically extracted |
| **Certainty of evidence** | 22 | Present assessments of certainty (or confidence) in the body of evidence for each outcome assessed. | Results - Table 1 (pp. 6-23) and Discussion: OCEBM levels of evidence presented for each study (Level 1b through Level 4). Overall certainty assessed as LOW to MODERATE due to: (1) lack of randomized controlled trials, (2) small sample sizes particularly in intervention studies, (3) heterogeneous definitions and diagnostic criteria, (4) cross-sectional design predominance, (5) incomplete outcome reporting. Discussion concludes: "Heterogeneity in CSA definitions, diagnostic protocols, and outcome measures limits firm conclusions. Standardized criteria and adequately powered prospective studies are needed" |
| **DISCUSSION** |  |  |  |
| **Discussion** | 23a | Provide a general interpretation of the results in the context of other evidence. | Discussion section (pp. 26-27): Results interpreted as demonstrating: (1) CSA substantially more frequent post-stroke (8-12%) than general population (<1%), (2) CSA represents marker of high-risk cardio-cerebrovascular profile with disturbed central ventilatory control, (3) complex bidirectional relationship between CSA and stroke, (4) pathophysiology multifactorial combining focal lesions and cardiac dysfunction, (5) inconsistent lesion-CSA associations suggest stroke may unmask pre-existing CSA rather than cause it. Placed in context of OSA literature, stroke rehabilitation guidelines, and cardiac disease management |
|  | 23b | Discuss any limitations of the evidence included in the review. | Discussion section (pp. 26-27): Extensive discussion of evidence limitations: (1) heterogeneous CSA definitions (CAI cutoffs vary from ≥5 to ≥50 events/hour; pattern-based definitions), (2) diagnostic method variation (polysomnography vs. portable vs. home testing), (3) inconsistent lesion-CSA associations, (4) no randomized controlled trials for interventions, (5) small sample sizes particularly for ASV studies, (6) selective sampling (severe strokes excluded), (7) heterogeneous outcome measures, (8) insufficient evidence that treating CSA changes outcomes, (9) directional associations incompletely defined (CSA as cause vs. marker), (10) sex differences and very elderly underrepresented, (11) small-vessel vs. large-artery stroke differences unclear |
|  | 23c | Discuss any limitations of the review processes used. | Discussion section (pp. 26-27): Limitations of review processes addressed: (1) gray literature and non-peer-reviewed publications excluded, (2) search restricted to English and Spanish language publications (potentially missing non-English publications), (3) risk of bias assessment limited to OCEBM level of evidence rather than formal structured tool, (4) meta-analysis not feasible due to heterogeneity, (5) no formal publication bias assessment, (6) potential for reviewer bias in study selection and data extraction despite dual-reviewer approach, (7) cannot exclude studies selected based on positive findings (selective outcome reporting bias not formally assessed) |
|  | 23d | Discuss implications of the results for practice, policy, and future research. | Discussion section (pp. 26-27) and Conclusions (p. 27): Practice implications: (1) CSA detection should prompt evaluation for coexisting cardiac dysfunction, (2) central patterns often improve spontaneously over months, (3) management should be individualized and dynamic, (4) therapeutic decisions depend on predominant type and severity of sleep apnea, (5) CPAP standard for obstructive patterns; ASV for predominantly central events; non-invasive ventilation for hypercapnic CSA, (6) current practice largely expert opinion rather than robust evidence. Policy implications: European guidelines encourage systematic screening/treatment in acute stroke; American guidelines do not mandate routine screening, reflecting uncertainty. Future research: (1) Standardized CSA definitions and diagnostic protocols needed, (2) Adequately powered prospective longitudinal studies from acute to chronic phases, (3) Mechanistic research clarifying directionality and causality, (4) Pragmatic intervention trials examining impact of treating CSA on long-term survival and recurrent stroke risk, (5) Investigation of sex differences and very elderly populations, (6) Studies comparing small-vessel vs. large-artery stroke phenotypes |
| **OTHER INFORMATION** |  |  |  |
| **Registration and protocol** | 24a | Provide registration information for the review, including register name and registration number, or state that the review was not registered. | Materials and Methods section (p. 3): Study protocol registered with Open Science Framework (OSF) under identifier DOI: 10.17605/OSF.IO/H59RJ |
|  | 24b | Indicate where the review protocol can be accessed, or state that a protocol was not prepared. | Materials and Methods section (p. 3): Protocol accessible via OSF registration link (DOI: 10.17605/OSF.IO/H59RJ). Review conducted according to PRISMA extension for scoping reviews (PRISMA-ScR) following predetermined protocol |
|  | 24c | Describe and explain any amendments to information provided at registration or in the protocol. | Not explicitly described in document. No amendments mentioned |
| **Support** | 25 | Describe sources of financial or non-financial support for the review, and the role of the funders or sponsors in the review. | "Funding" section (p. 28): "This research received no external funding." Therefore, no financial or non-financial support sources to declare |
| **Competing interests** | 26 | Declare any competing interests of review authors. | “Conflicts of Interest" section (p. 28): "The authors declare there are no conflicts of interest." |
| **Availability of data, code and other materials** | 27 | Report which of the following are publicly available and where they can be found: template data collection forms; data extracted from included studies; data used for all analyses; analytic code; any other materials used in the review. | "Availability of Data and Materials" section (p.27): "The datasets generated and analyzed during this scoping review are available from the corresponding author on reasonable request." Data collection form referenced as Supplementary Material II (customized Microsoft Excel-based extraction form); search strategy provided in Supplementary Material I |

*From:* Page MJ, McKenzie JE, Bossuyt PM, Boutron I, Hoffmann TC, Mulrow CD, et al. The PRISMA 2020 statement: an updated guideline for reporting systematic reviews. BMJ 2021;372:n71. doi: 10.1136/bmj.n71. This work is licensed under CC BY 4.0. To view a copy of this license, visit https://creativecommons.org/licenses/by/4.0/
